# Epstein-Barr virus infectious particles initiate B cell transformation and modulate cytokine response

**DOI:** 10.1128/mbio.01784-23

**Published:** 2023-10-13

**Authors:** Francesco Baccianti, Charlène Masson, Susanne Delecluse, Zhe Li, Remy Poirey, Henri-Jacques Delecluse

**Affiliations:** 1 Pathogenesis of Virus Associated Tumors, German Cancer Research Center (DKFZ), Heidelberg, Germany; 2 Unit U1074, INSERM, Heidelberg, Germany; 3 Nierenzentrum Heidelberg e.V., Heidelberg, Germany; 4 Deutsches Zentrum für Infektionsforschung (DZIF), Braunschweig, Germany; Columbia University Medical College, New York, New York, USA

**Keywords:** Epstein-Barr virus, STAT3, p38-MK2, ZFP36L1

## Abstract

The Epstein-Barr virus (EBV) efficiently transforms primary B cells. Here, we show that this process starts immediately after cellular exposure to infectious viral particles. Virus binding to B cells led to the activation of intracytoplasmic tyrosine kinases and STAT3. Tegument proteins within the virion in turn activated the p38-MK2 pathway upon cell entry, independently of the viral DNA. Engagement of STAT3 and p38/MK2, two pro-inflammatory pathways, was essential for expression of the key EBV transforming gene EBNA2 but also facilitated IL-6 and TNFα release. However, these pathways simultaneously activated ZFP36L1, a stress response protein that targets transcripts with an AU-rich 3′UTR, to reduce IL-6 and TNFα transcription in infected cells. Expression of viral latent proteins after infection amplified the viral effects on p38 and MK2, but also on ZFP36L1, altogether resulting in a transitory and limited increase in IL-6 and TNFα transcription and release. Thus, EBV virions are not merely vehicles that allow injection of the viral DNA into the nucleus but manipulate cellular pathways to initiate transformation while limiting cytokine release.

**IMPORTANCE:**

The Epstein-Barr virus efficiently infects and transforms B lymphocytes. During this process, infectious viral particles transport the viral genome to the nucleus of target cells. We show here that these complex viral structures serve additional crucial roles by activating transcription of the transforming genes encoded by the virus. We show that components of the infectious particle sequentially activate proinflammatory B lymphocyte signaling pathways that, in turn, activate viral gene expression but also cause cytokine release. However, virus infection activates expression of ZFP36L1, an RNA-binding stress protein that limits the length and the intensity of the cytokine response. Thus, the infectious particles can activate viral gene expression and initiate cellular transformation at the price of a limited immune response.

## INTRODUCTION

The Epstein-Barr virus (EBV) infects the large majority of the world’s adult population (1) and is associated with the development of several malignancies, mainly lymphomas such as Burkitt’s lymphomas (BL), but also carcinomas of the stomach and of the nasopharynx (1). Furthermore, EBV plays a crucial role in the development of multiple sclerosis (2, 3). In healthy humans, EBV mainly if not exclusively infects primary B cells (4). The virus first binds to the B cell surface molecule CD21, leading to its internalization in endosomal vesicles (5, 6). After fusion of the viral envelope with these vesicles, the viral capsid is extruded into the cytoplasm and transported to the nucleus to which it is tethered to allow injection of the viral genome 60 minutes after infection (6). During this process, viral proteins located in the tegument, a virtual space between the capsid and the viral envelope, are delivered to the cytoplasm of the infected cell (7). Six hours post-infection, primary B cells have already initiated the sequential expression of a set of eight viral genes that induce continuous B cell proliferation 6 to 7 days later, a process also known as latency 3 or active latency (8, 9). These so-called latent genes consist of the Epstein-Barr virus nuclear antigens (EBNAs) and of the latent membrane proteins (LMPs). EBNA2 is the cornerstone of this process as it transactivates the expression of both cellular and viral latent genes, including the LMP1 protein that is endowed with oncogenic properties (10, 11). EBNA gene expression is initially driven by the Wp promoter that is fully active 6 hours after viral infection (8, 12, 13). Approximately 2 days later, the transcriptional activity of the Wp promoter is taken over by the adjacent Cp promoter (14, 15). In contrast to Wp, Cp is transactivated by EBNA2. During the early stages of infection, cellular signaling pathways are also engaged. STAT3 activation has been shown to downregulate the DNA damage response and to facilitate progression through the cell cycle. STAT3 impairs ATR-mediated phosphorylation of Chk through the sequential activation of caspases. This prevents activation of the intra-S phase checkpoint in response to the viral-induced cellular replication stress, allowing cell cycle progression (16 – 18).

For many intracellular pathogens, the very early stages of infection are also essential to downregulate the innate immune response that would otherwise block the incoming viral particles, thereby preventing the establishment of the infection (19, 20). This includes the modulation of signaling pathways by viruses to prevent the activation of pattern recognition receptors (PRR) or to counteract their effects (21). The inflammatory response that follows PRR activation after infection represents a double-edged sword for the virus as some cytokines facilitate infection, yet others block it (22 – 24). The NF-κB pathway is thought to control the inflammatory process that signals downstream the majority of PRR and intracellular sensing pathways (25). Indeed, NF-κB was initially reported to control the secretion of the pro-inflammatory cytokines interleukin 6 (IL-6) and tumor necrosis factor alpha (TNFα) observed after EBV infection of B cells (26, 27). However, more recent lines of evidence have suggested that several EBV proteins can restrict the activation of the NF-κB pathway during the early stages of the infection (28 – 31). Accordingly, NF-κB activation is complete only when high and stable expression of LMP1 is achieved (32, 33).

Despite their significance in the EBV life cycle, early events following infection have not yet been thoroughly investigated. Most studies have focused on events occurring after the inception of the EBV active latency program (34 – 36). Consequently, we conducted high-throughput screens to identify the molecular events that regulate the initial hours of the infection process. Using this approach, we discovered that the two pro-inflammatory signaling pathways STAT3 and p38-MK2-ZFP36L1 are activated during virus binding and cell entry. Activation of these pathways was necessary for viral latent gene expression and the initiation of B cell transformation, at the price of a limited pro-inflammatory response with cytokine release.

## RESULTS

### Early molecular events after EBV primary B cell infection

**Fig 1:**
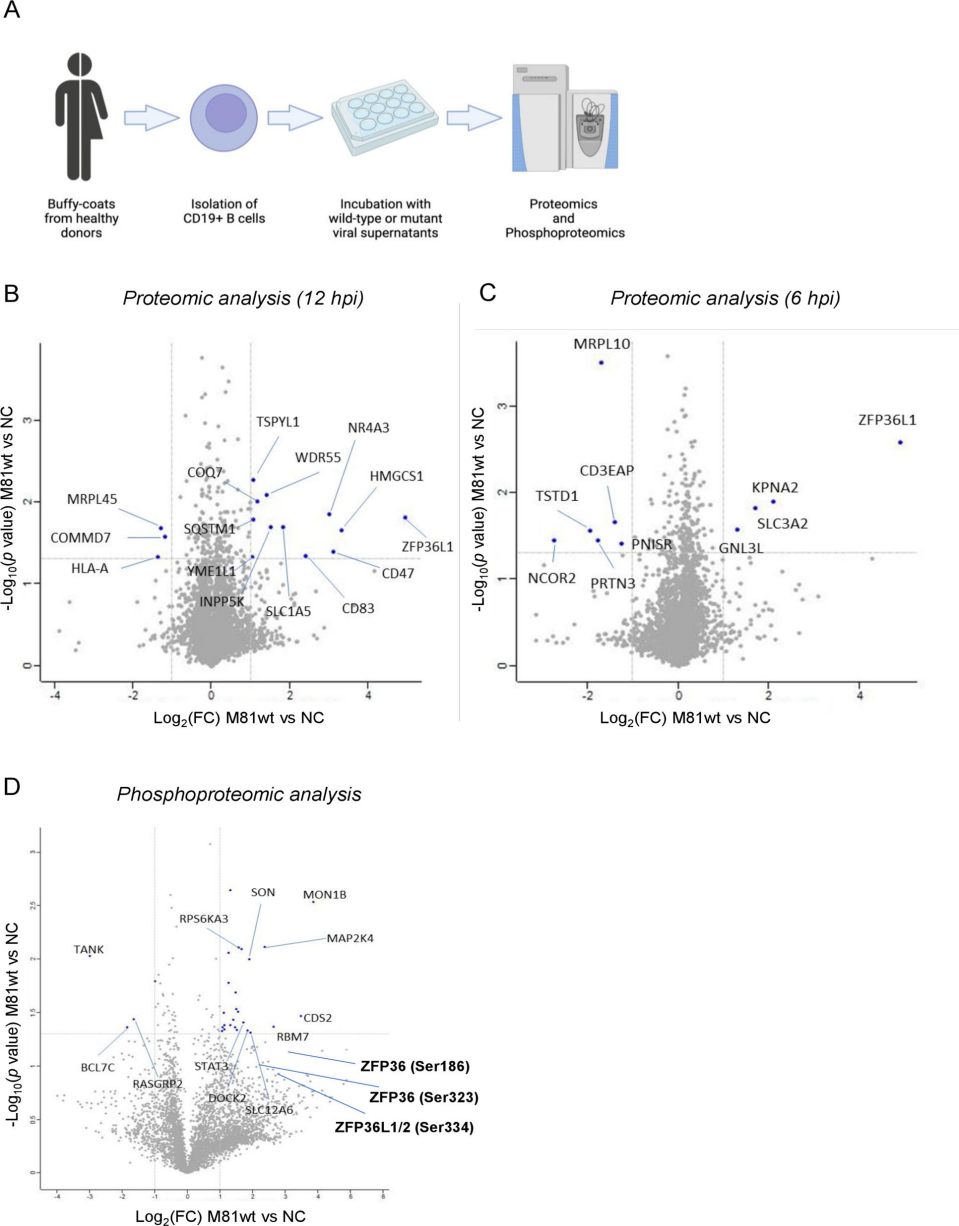
Proteomic and phosphoproteomic analysis performed on EBV M81wt-infected cells early after infection. (**A**) Overview of the workflow. (**B–C**) Volcano plot representation of significantly upregulated and downregulated proteins as identified by label-free mass spectrometry performed on two independent biological samples of primary human CD19^+^ B cells at 12 hours (**B**) and at 6 hours (**C**) post-infection with M81wt virus. (**D**) Volcano plot representation of significantly upregulated and downregulated phosphopeptides as identified by phosphoproteomic analysis in three independent biological pooled replicates of primary human CD19^+^ B cells treated with M81wt at 6 hours post-infection. (**B–D**) A two-tailed paired *t*-test was performed in both cases. Targets were selected if their *P* value was ≤0.05 (horizontal dotted line) and the absolute fold change versus the negative control was >2 (vertical dotted lines). Selected candidates for which the *P* value is above significance are shown in bold.

To identify early molecular events after EBV infection of primary B cells, we performed a high-throughput analysis of their proteome and phosphoproteome profile 6 and 12 hours post-EBV infection (Fig. 1A). In two independent primary B cell samples, we found 12 proteins that were upregulated and 3 that were downregulated 12 hours after wild-type infection (Table 1; Fig. 1B). Pathway analyses of these data did not reveal any specific signature, in particular no interferon response. Quantification of interferon alpha release using enzyme-linked immunosorbent assay (ELISA) at various time points after infection showed that secretion of this cytokine began after 16 hours and reached significant levels only 96 hours after infection (Fig. S1A). Among the proteins identified on the screen, ZFP36L1 (also known as TIS11B or BRF1) was consistently upregulated already at 6 hours post-infection (Fig. 1C). We then exposed B cells to an EBV M81/∆gp110 knockout that lacks the fusion protein gp110 or to EBV virus-like particles (VLP) that enter B cells but are devoid of DNA (Fig. S1B and C). The former virus can bind to B cells but cannot infect them, while the latter cannot establish a chronic virus infection (37, 38). Proteomic analysis of these samples showed that ZFP36L1 was clearly upregulated after exposure to EBV VLPs, but not to wild-type levels. In contrast, the effect on ZFP36L1 was only marginal after M81/∆gp110 binding ( Fig. S1D). The generated data also confirmed recently identified cellular targets of the virus. At 6 hours post-infection, we observed a significant reduction in SMC6 protein expression after B cell exposure to both wild-type virus and VLPs, but not after incubation with the ∆gp110-defective mutant (Fig. S1E) (39). Moreover, we detected a modest but statistically significant reduction in the levels of STAT2 protein after M81wt infection (Fig. S1F) (40).

**TABLE 1:** List of targets which are up- or downregulated (absolute fold change > 2) and statistically significant (*P* ≤ 0.05) at 12 hours post-infection

Gene names	*P* value M81wt_NC (12 hpi)	Fold change M81wt_NC (12 hpi)
ZFP36L1	0.02	30.97
HMGCS1	0.02	9.95
CD47	0.04	8.68
NR4A3	0.01	8.06
CD83	0.04	5.32
SLC1A5	0.02	3.57
INPP5K	0.02	2.84
WDR55	0.01	2.64
COQ7	0.01	2.25
SQSTM1	0.02	2.10
TSPYL1	0.01	2.08
YME1L1	0.05	2.06
COMMD7	0.03	2.28
MRPL45	0.02	2.45
HLA-A	0.05	2.60

A phosphoproteomic analysis performed on B cells infected with wild-type virus revealed multiple phosphoevents in the STAT3 protein, in members of the mitogen-activated protein kinase (MAPK) pathway, and in the RNA-binding proteins RMB7 and ZFP (ZFP36, ZFP36L1, and ZFP36L2) (Fig. 1D; Table S1). However, in the latter case, although the recorded values were all indicative of an increase in phosphoevents, the global analysis did not reach statistical significance due to a high level of standard deviation between recorded values. Similar experiments performed with EBV VLPs led to the detection of more phosphoevents than after wild-type infection, suggesting that some initial kinase targets of the virus revert at a later stage of infection ( Fig. S2A). Binding of M81/∆gp110 also generated phosphoevents, some of which were common to those generated by EBV VLP and wild-type infection (Fig. S2B and C; Table S1). In particular, phosphoevents in members of the ZFP36 family (41) were common to all types of infection (Fig. S2D). Pathway analysis on the list of up- or downregulated phosphoevents revealed enrichment of MAPK and p38 after exposure of B cells to VLP and suggested their activation. A similar trend that did not reach statistical significance was observed in B cells infected with M81wt or M81/∆gp110 (Fig. S2E).

Altogether, these high-throughput screens identified an upregulation and phosphorylation of ZFP36L1, together with STAT3 phosphorylation as important early events after EBV infection. These events were already present after virus binding.

### EBV binding activates STAT3

**Fig 2:**
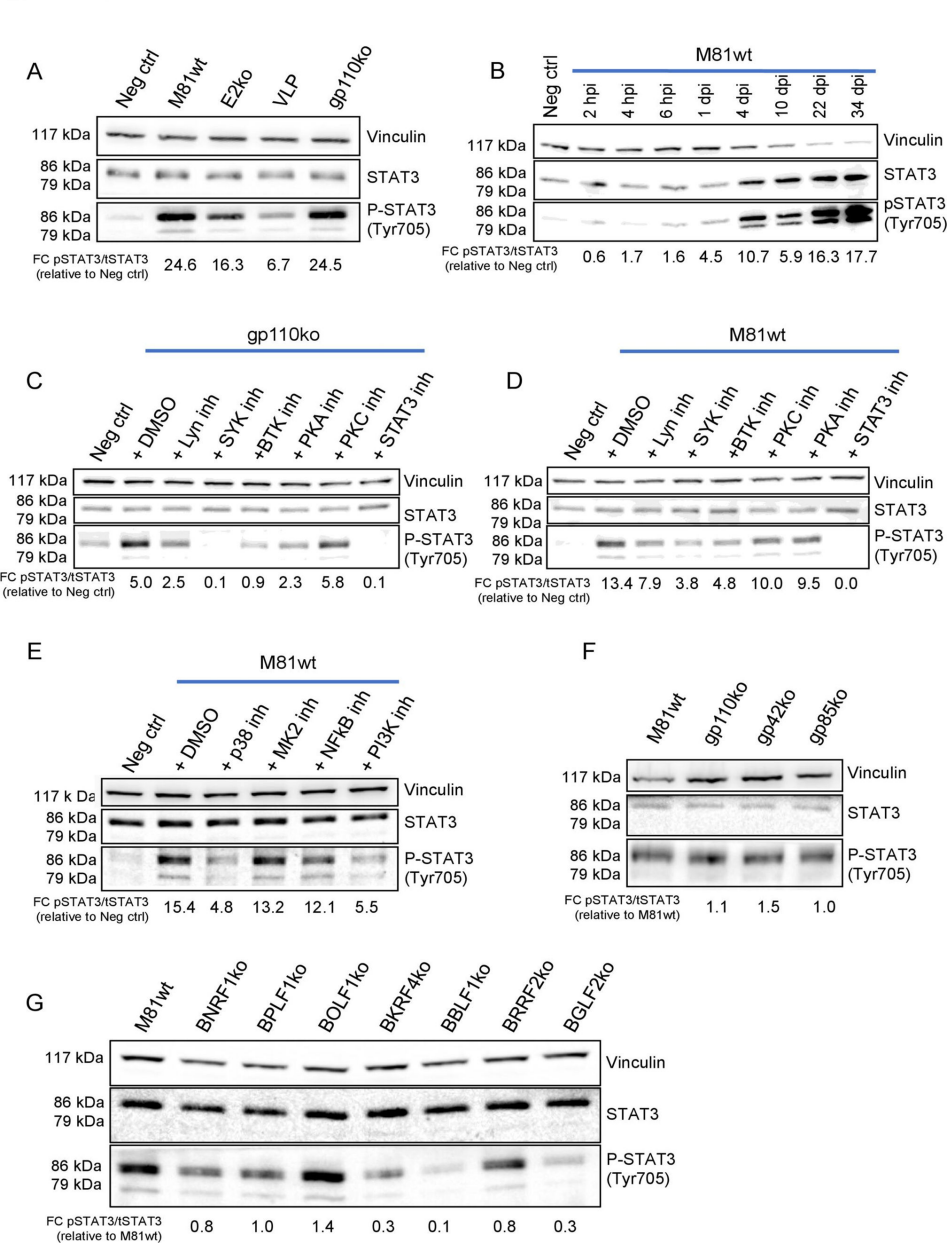
STAT3 activation occurs upon EBV binding. Western blot analysis for activated STAT3 (Tyr705) was performed on human primary CD19^+^ B cells (**A**) infected with M81wt, EBNA2 knockout, M81 VLPs, and gp110 knockout viruses at 6 hours post-infection; (**B**) infected with M81wt virus at different time points post-infection; exposed to the gp110 knockout virus (**C**) or infected with M81wt virus (**D**) in the presence of inhibitors of B cell receptor signaling downstream mediators; (**E**) infected for 6 hours with M81wt virus in the presence of p38, MK2, PI3K, and NF-κB pathway inhibitors; (**F**) exposed to M81wt gp110, gp85, and gp42 knockout viruses; and (**G**) infected with different M81 tegument knockouts. (**A–G**) Total STAT3 and phospho-STAT3 (Tyr705) were detected, and vinculin was used as a loading control. Each blot is representative of at least three biological replicates, and the reported values represent the fold change (FC) versus the controls for the indicated normalized ratios.

We could confirm STAT3 activation suggested by the phosphoproteome by performing western blot with a p-STAT3- (Tyr705) specific antibody on EBV-infected B cell samples. We used a panel of viruses that included wild-type virus, its EBNA2 deletion mutant (Fig. S1B and C), VLPs, or M81/∆gp110 to perform infections. M81/∆EBNA2 can infect B cells but cannot initiate active latency. While all infected samples expressed p-STAT3, their total STAT3 levels remained largely unchanged (Fig. 2A; Fig. S3A). However, infection with the EBNA2 deletion mutant or with VLPs was less efficient at inducing p-STAT3 than infection with M81 wt or with M81/∆gp110. Cell fractionation showed that the increase in pSTAT3 upon EBV infection is mainly concentrated in the nucleus (Fig. S3B). Monitoring pSTAT3 levels over time after wild-type infection revealed that phosphorylation of STAT3 began as early as 45 minutes after infection and continuously increased until it reached a plateau between 6 and 24 hours post-infection (Fig. 2B; Fig. S3C). STAT3 activation continued to increase after 24 hours post-infection and reached a maximum by 22 days post-infection (Fig. 2B). These results are in line with the observation that STAT3 is upregulated in established LCLs under the influence of LMP1 (42). Infection with M81/∆gp110 strongly increased p-STAT3 levels, suggesting that this event occurs upon virus binding. Indeed, preincubation of wild M81 viruses with AMMO1, an antibody that blocks viral fusion, but not binding, also activated STAT3 (Fig. S3D) (43). We further confirmed that the observed effects were due to the infectious particles themselves, as the conditioned medium was unable to induce STAT3 phosphorylation (Fig. S3D).

We also studied the effects of treatment with bafilomycin A on infection. Bafilomycin A, a known inhibitor of endosomal trafficking, halved phospho-STAT3 levels, compared with the infection with vehicle alone (Fig. S3D). However, phosphorylated STAT3 levels have been shown to be significantly affected by the V-ATPase inhibitor activity of bafilomycin A which promotes dephosphorylation of STAT3 and relocalization of unphosphorylated STAT3 to the lysosomal compartment (44). This suggests that the effect on infection observed upon bafilomycin A treatment is not attributable to the inhibition of endosomal trafficking, but rather to the dephosphorylation and relocalization of STAT3 induced by the inhibitor.

We then attempted to identify intracellular kinases involved in STAT3 Tyr705 phosphorylation. STAT3 is typically phosphorylated at Tyr705 by the JAK pathway (45, 46). However, treatment with AG490, a selective inhibitor of JAK2, only marginally reduced STAT3 phosphorylation levels upon M81 infection (Fig. S3E). We exposed primary B cells to M81/∆gp110 virus in the presence of pathway inhibitors that selectively target mediators downstream of the B cell receptor (BCR) signaling platform. In resting B cells, the BCR plays a pivotal role in transducing the signal initiated upon engagement by antigens (47, 48). Furthermore, CD21, the principal cellular receptor for gp350 in primary B cells, is able to mediate signal transduction, either directly or indirectly via components of the BCR signaling cascade (49, 50). Inhibition of SYK and BTK produced the strongest reduction in STAT3 Tyr705 phosphorylation levels, while LYN and PKA inhibition had a more limited effect (Fig. 2C; Fig. S3E). Similar experiments using wild-type virus confirmed the importance of SYK for STAT3 activation, although its inhibition did not completely block STAT3 phosphorylation (Fig. 2D). P-STAT3 levels were decreased after inhibition of p38 and PI3K (Fig. 2E). To confirm the results obtained with M81/∆gp110, we exposed primary B cells to M81/∆gp85 and M81/∆gp42 knockout viruses and assessed their ability to activate STAT3. These viruses retain full binding activity (see Fig. S4A and B) but are unable to fuse with their target cells (51). This experiment showed that viral proteins involved in virus-cell fusion are not necessary for STAT3 phosphorylation (Fig. 2F). We completed this analysis by infecting B cells with a panel of viruses lacking one of the main EBV tegument proteins. All mutants efficiently bound to their target cells as determined by flow cytometry using a gp350-specific antibody (Fig. S4C and D). Only BBLF1, BGLF2, and BKRF4 were found to be required for full STAT3 activation (Fig. 2G). We conclude that STAT3 is phosphorylated by SYK as the result of virus binding but that p-STAT3 levels are further modulated by downstream events which follow virus entry and in which both tegument proteins and the establishment of latency play a role.

### EBV virus-like particles are sufficient to activate ZFP36L1 expression

**Fig 3:**
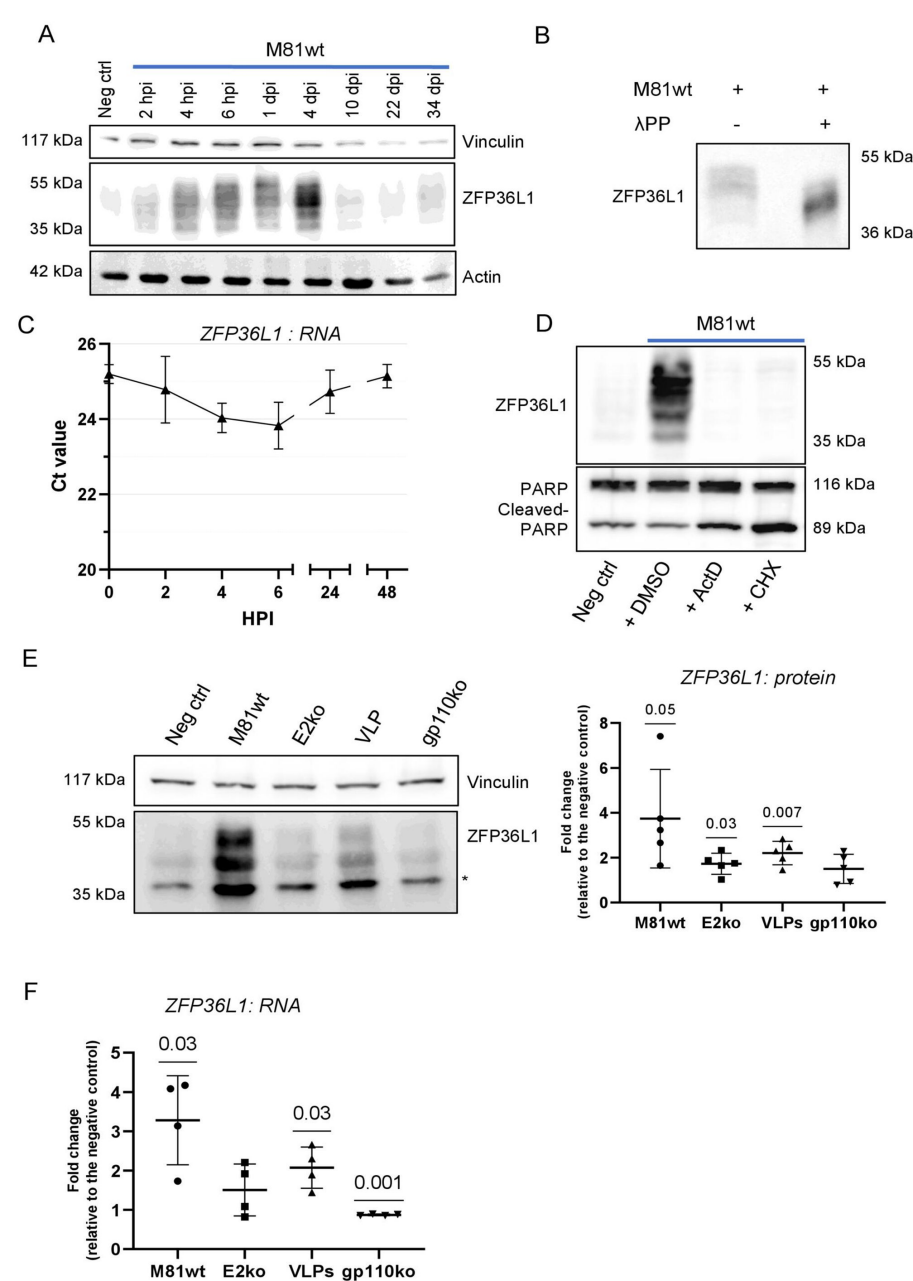
ZFP36L1 is highly upregulated upon EBV infection. (**A**) ZFP36L1 expression upon EBV infection as shown by western blot. A time-course experiment was performed on primary human B cells infected with M81wt. ZFP36L1 was detected in samples obtained at the indicated time point post-infection. (**B**) Cell lysates from M81wt-infected B cells at 6 hours post-infection were treated, or not, with λ protein phosphatase and immunoblotted for ZFP36L1. (**C**) ZFP36L1 mRNA quantification by RT-qPCR at different hours post-M81wt virus infection. Ct values from three independent biological replicates are represented as mean ± SD. (**D**) Western blot analysis of primary human B cells infected with M81wt in the presence of the vehicle dimethylsulfoxide (DMSO) only or actinomycin D (ActD), a transcriptional inhibitor, or cycloheximide (CHX), a translational repressor. The detection was performed using antibodies against ZFP36L1 and PARP/cleaved-PARP to assess toxicity. (**E**) ZFP36L1 protein expression levels were detected by western blot after infection with M81wt, EBNA2 knockout, M81 VLPs, and gp110 knockout virus at 6 hours post-infection. In the right panel, the signal for ZFP36L1 was quantified and normalized over the respective loading control. The fold change was calculated versus the negative control. The graph reports the values as the mean ± SD (*n* = 5 independent biological replicates). (**F**) ZFP36L1 RNA expression levels were quantified via RT-qPCR at 6 hours post-infection with M81wt, EBNA2 knockout, VLPs, or gp110 knockout virus and represented as fold change versus the negative control. The difference for each independent replicate is plotted, and the mean ± SD is reported (*n* = 4 independent biological replicates). (**E and F**) A one-sample *t*-test (*μ* = 1) was performed. *P* values are reported above the comparison. *P* ≤ 0.05 was considered statistically significant. * indicates phospho-p38 (**E**).

Western blot analyses confirmed that while ZFP36L1 was hardly present in resting B cells, its expression was upregulated 2 hours after EBV infection and that it persisted for several days (Fig. 3A). ZFP36L1 appearance as a ladder in western blot has previously been ascribed to different levels of phosphorylation (Fig. 3A) (52 – 56). We confirmed that this indeed is the case as the protein ladder reduced to a single band after treatment of the samples with a phosphatase (Fig. 3B) (57, 58). RT-qPCR-based analysis of transcription showed that the increase in ZFP36L1 protein production is paralleled by a short increase in transcription that reached a maximum at 6 hours post-infection but returned to baseline levels after 2 days (Fig. 3C). To investigate whether ZFP36L1 upregulation was the result of an increased *de novo* transcription or translation, we infected B cells with M81wt virus for 6 hours in the presence of the transcription inhibitor actinomycin D or of the translation inhibitor cycloheximide (Fig. 3D). This experiment clearly indicated that ZFP36L1 upregulation upon infection occurred as the result of increased *de novo* transcription and translation (Fig. 3D). The infection experiments were then extended to deletion mutants. This assay showed that VLPs or M81/∆EBNA2 less readily induced ZFP36L1, relative to wild-type infection (Fig. 3E). We also observed a very weak ZFP36L1 induction after M81/∆gp110 infection. We noted that the intensity of ZFP36L1 induction, as assessed by western blot, was lower than reported by the results of the proteomics analysis. This might be due to the quality of the ZFP36L1-specific antibodies used, but also to the multiple ZFP36L1 phosphorylation sites that are difficult to visualize and quantify. Transcription analyses revealed a similar pattern, with exposure to VLPs or M81/∆EBNA2 being less efficient at activating ZFP36L1 expression than wild-type M81 (Fig. 3F). Binding of M81/∆gp110 to B cells proved unable to activate ZFP36L1 transcription. Immunofluorescence stains for ZFP36L1 on freshly infected primary B cells showed increased ZFP36L1 expression upon EBV infection, with accumulation of the protein in the cytoplasm where the protein can access its RNA targets (Fig. S5A) (59 – 62). Here again, the wild-type virus proved to be more potent than the M81 VLPs or ∆gp110 in stimulating ZFP36L1 protein production. Altogether, these experiments confirmed proteomic data and showed that ZFP36L1 becomes increasingly activated as the infection progresses from virus binding to the inception of latent gene expression.

### Exposure of B cells to EBV induces MK2, the ZFP36L1 master regulator

**Fig 4:**
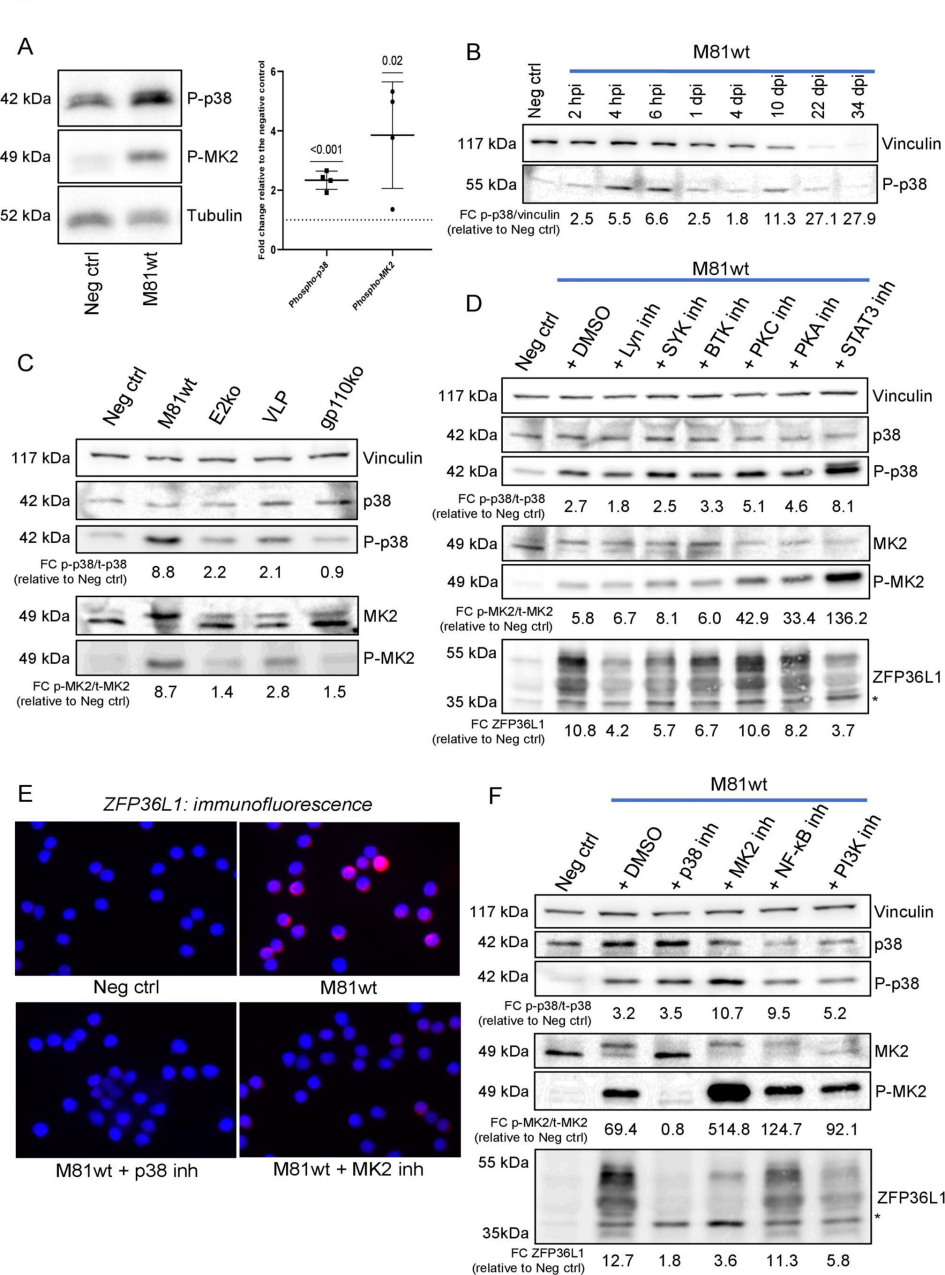
ZFP36L1 is expressed and phosphorylated upon EBV infection, a process dependent on the activation of p38/MK2. (**A**) EBV M81wt-infected B cells were analyzed at 6 hours post-infection for their levels of p38 and MK2 activation using immunoblotting for phosphorylated p38 (Thr180/Tyr182) and phosphorylated MK2 (Thr334), respectively. An example of a blot is shown (left panel). The intensity of the signal in the bands was quantified via ImageJ and normalized over the loading control. The fold change is calculated versus the uninfected control (*n* = 4 independent biological replicates). Single values are plotted, and the mean ± SD is shown (right panel). A one-sample *t*-test (*μ* = 1) was performed. *P* values are reported above the comparison. *P* ≤ 0.05 was considered statistically significant. (**B**) p38 activation was detected in M81wt-infected primary B cells at different time points after infection. (**C**) p38 and MK2 activation was detected via immunoblotting in human primary B cells at 6 hours post-infection with M81wt virus, EBNA2 knockout, M81 VLPs, or gp110 knockout. p38 and MK2, as well as their phosphorylated counterparts, were detected, and vinculin was used as a loading control. (**D**) The same panel of antibodies, together with an anti-ZFP36L1 antibody, was used to stain primary B cells infected with M81wt virus in the presence of inhibitors of the downstream mediators of the BCR signaling cascade. (**E**) Immunofluorescence staining for the detection of ZFP36L1 in human primary B cells infected with M81wt virus in the presence of p38 or MK2 inhibitors at 6 hours post-infection. ZFP36L1 signal is shown in red, while nuclei are counterstained with DAPI (blue). (**F**) Western blot analysis of p38/MK2 and ZFP36L1 expression in human primary B cells infected with M81wt virus and treated with p38, MK2, NF-κB, and PI3K pathway inhibitors. Where not indicated, each blot is representative of at least three biological replicates (*n* = 3). The fold change (FC) relative to the negative control for the total and phospho ratios for p38 and MK2, as well as for ZFP36L1 expression, are given. * indicates phospho-p38. The same set of biological samples was used to perform the experiments described in Fig. 2A, D, and E and in Fig. 4C, D, and E. Consequently, the loading control vinculin is identical for both set of figures.

Because MK2 was previously reported to regulate the expression and phosphorylation of the ZFP36 protein family members to which ZFP36L1 belongs, we used phospho-specific antibodies to detect activation of MAPK p38 and of its downstream target MK2 (Fig. 4A) (53, 63 – 65). This assay confirmed activation of MAPK p38 and MK2 upon infection. P-p38 was already detectable 2 hours post-infection and reached its maximum at 6 hours (Fig. 4B). The accumulation of phosphorylated p38 was not paralleled by an increase in total p38 and did not result in the activation of other downstream branches of the p38 pathway such as MSK2 or ATF2 (Fig. S5B). While p38 activation and ZFP36L1 expression simultaneously increased after B cell infection, phospho-p38 already returned to baseline levels after 1 day (Fig. 3A and 4B). Exposure of primary B cells to EBV VLPs or M81/∆EBNA2 also led to p38 induction, although it could not reach levels seen after wild-type infection (Fig. 4C). Similarly, MK2 activation was weaker after infection with EBV VLPs or with the EBNA2-null virus. Infection with the gp110-null mutant did not activate the p38/MK2 pathway at all (Fig. 4C). We then assessed the phosphorylation patterns of PI3K-AKT and ERK1/2, two other major B cell signaling pathways, and did not observe any significant changes (Fig. S5C). Since NF-κB was previously reported to be activated after early EBV infection events, we investigated the activation status of its canonical branch (27, 66). Phospho-p65 and IkBa expression was already observed in resting B cells and did not consistently increase upon infection (Fig. S5D). Inhibitors of SYK, LYN, and BTK did not influence p38 or pMK2 expression and their activation (Fig. 4D). Interestingly, treatment with PKA inhibitor H-89, PKC inhibitor BIM I, and STAT3 inhibitor Stattic modified the levels of total p38 and MK2. Stattic also increased the levels of phosphorylation of the two proteins, a phenomenon previously observed by Guha and colleagues which indicates a possible interplay between these two pathways (67). The impact of this panel of inhibitors on ZFP36L1 was broader, with the LYN, SYK, BTK, and STAT3 inhibitors leading to a significant decrease in ZFP36L1 abundance. As anticipated, the MK2 and p38 inhibitors markedly reduced ZFP36L1 transcription, protein expression, and phosphorylation (Fig. 4E and F; Fig. S5E). This confirms the importance of the p38 pathway in controlling ZFP36L1 expression levels but also shows that other kinases modulate its abundance and phosphorylation pattern.

### EBV infection leads to a two-step activation of IL-6 and TNFα release

**Fig 5:**
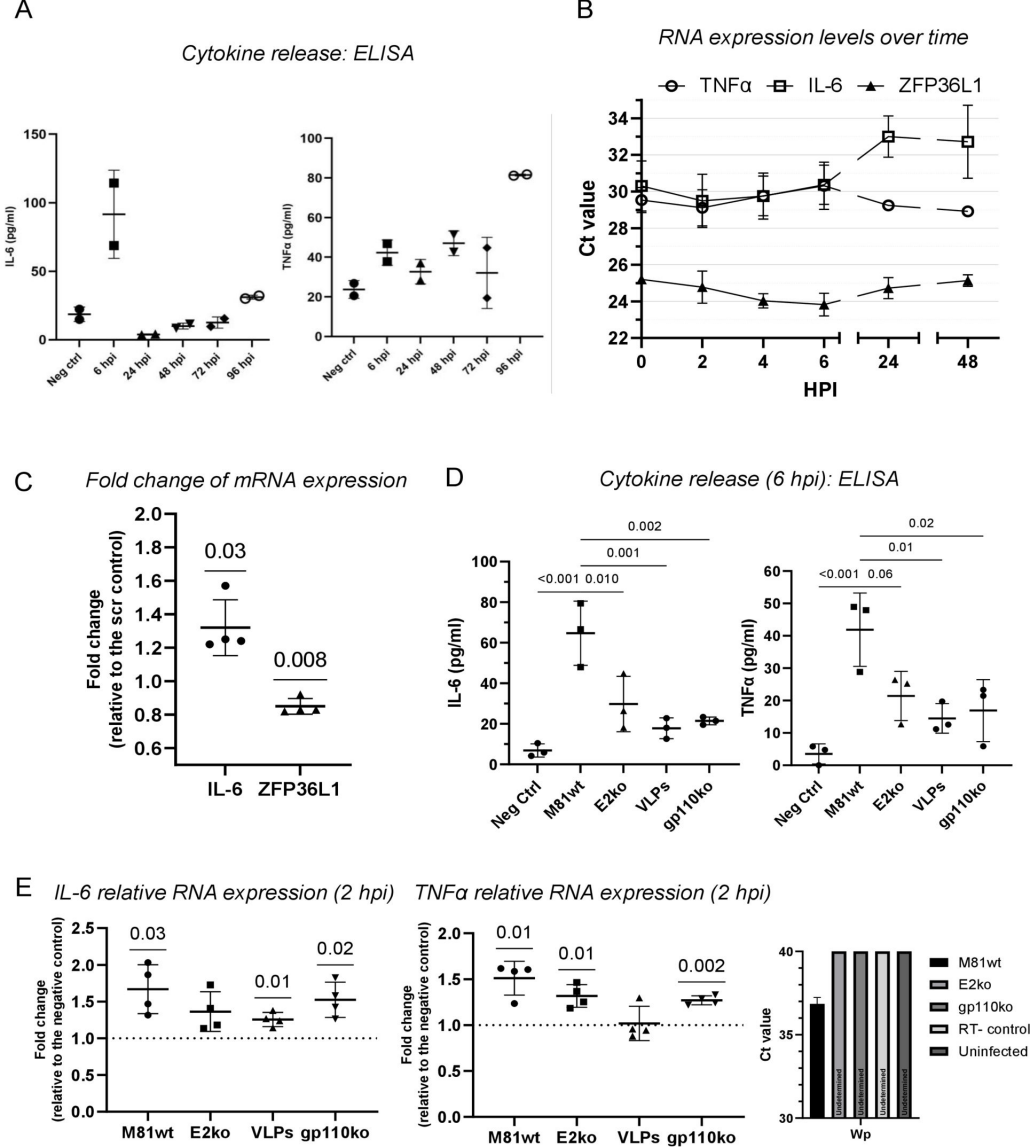
EBV infection induces IL-6 and TNFα secretion in two separate steps. (**A**) IL-6 and TNFα secretion by primary human CD19^+^ B cells infected with M81wt virus. At each time point, cells were seeded at the same cell concentration and incubated for 6 hours. The release of the two cytokines was measured by ELISA. Single values were plotted, and the mean ± SD is shown (*n* = 2 independent biological replicates). (**B**) IL-6, TNFα, and ZFP36L1 mRNA expression as quantified by reverse transcription quantitative PCR at different time points upon infection with M81wt virus. Ct values are reported as the mean ± SD (*n* = 3 independent biological replicates). (**C**) siRNA knock-down was performed before primary B cells were infected with M81wt virus. mRNA levels of IL-6 and ZFP36L1 are expressed as fold change relative to the negative (scr) control. *n* = 4 independent biological replicates. (**D and E**) IL-6 and TNFα, protein secretion, and mRNA expression as quantified by ELISA (**D**) at 6 hours post-infection and RT-qPCR (E, left and center) at 2 hours post-infection with M81wt virus, EBNA2 knockout, M81 VLPs, and gp110 knockout virus. Values obtained from three (**D**) or four (**E**) independent primary samples are reported, together with the mean ± SD. For the mRNA quantification, values are shown as the fold change versus the negative control. The level of activation of the Wp promoter was measured by RT-qPCR in the same donors, and the Ct values are reported (E, right). Undetermined indicates the lack of amplification in the sample. A one-way ANOVA with Tukey’s post hoc test (**D**) or a one-sample *t*-test (*μ* = 0) (**C, E**) was performed. *P* values are reported above the comparison. *P* ≤ 0.05 was considered statistically significant.

The ZFP36 family members bind to RNAs carrying an AU-rich stretch in their 3′UTR and inactivate them through a combination of decapping and deadenylation (68). In particular, TNFα and IL-6 mRNAs have been shown to be regulated by these proteins (69 – 71). This is interesting as both cytokines are released upon virus binding to B cells (26, 72). Indeed, quantification of cytokine production by ELISA revealed a spike in secretion 6 hours post- infection with a doubling in TNF alpha levels and a fivefold increase in IL-6 levels (IL-6 average 90 pg/mL, TNFα 40 pg/mL) that were already back to baseline levels 1 day after infection (Fig. 5A). However, these levels were much lower than those observed after treatment of infected B cells with TPA or LPS (Fig. S5F). This suggests that virus infection did not cause maximum cytokine release. Moreover, while IL-6 and TNFα transcripts tripled in numbers within 2 hours after the infection, they returned to baseline levels within 6 hours (Fig. 5B). IL-6 transcripts further diminished in abundance after 24 hours, but TNF alpha transcripts increased again at that time point. ZFP36L1 transcripts evolved exactly in the opposite direction, increasing when TNFα transcripts decreased and vice versa. In order to functionally validate ZFP36L1’s role in regulating IL-6 transcript stability upon infection, we knocked down ZFP36L1 expression in primary B cells with a specific siRNA prior to EBV infection (Fig. 5C). This resulted in a statistically significant decrease in ZFP36L1 transcripts coupled with an increase in the abundance of IL-6 transcripts.

We then repeated the cytokine induction experiment with B cells infected with M81/∆EBNA2, EBV VLPs, or the M81/∆gp110 virus. Quantification of cytokine release showed that exposure to EBV VLPs or to the M81/∆gp110 virus doubled on average cytokine release relative to uninfected cells but remained three times lower than after wild-type infection. Exposure of B cells to M81/∆EBNA2 resulted in intermediate cytokine release levels, reaching 50% of wild-type levels (Fig. 5D). Analysis of cytokine transcript levels 2 hours post-infection showed a similar pattern, with EBV VLP infection giving rise to a lower expression than wild-type infection and infection with M81/∆EBNA2 or M81/∆gp110 delivering intermediate results (see Fig. 5E and 3F for comparison with ZFP36L1 expression). We conclude that virus binding gives rise to a limited IL-6 and TNFα transitory release and that both the presence of the viral DNA within infectious particles and latent gene expression are required to reach maximal cytokine transcription and release, a process that started 2 hours post-infection. This correlated with the detection of rare Wp-driven latent transcripts already at this time point (Fig. 5E).

We then used our panel of pathway inhibitors to assess the contribution of the signaling pathways to IL-6 and TNFα secretion. When B cells were infected with the wild-type virus in the presence of inhibitors of BCR-associated kinases, both IL-6 and TNFα were variably affected, with the SYK inhibitor producing the strongest effect (Fig. S6A). Interestingly, the inhibitory effect was different for the two cytokines, pointing toward different regulatory mechanisms. We observed a significant reduction in both IL-6 and TNFα secretion when cells were treated with p38, MK2, and PI3K inhibitors, but only a limited effect was detected by inhibiting NF-κB (Fig. S6B). Similar effects on IL-6 and TNFα release were observed when B cells were exposed to M81/∆gp110 in the presence of BCR signaling inhibitors (Fig. S6C). In this context, the already low level of cytokine expression occurring upon M81/∆gp110 binding was nearly completely abolished by the SYK inhibition. Altogether, these results show that cytokine release after EBV infection is limited both in time and intensity, results from both virus binding and post-binding events including latency activation, and is controlled by signaling pathways activated at the early steps of the infection.

### EBV tegument proteins induce MK2 and ZFP36L1

**Fig 6:**
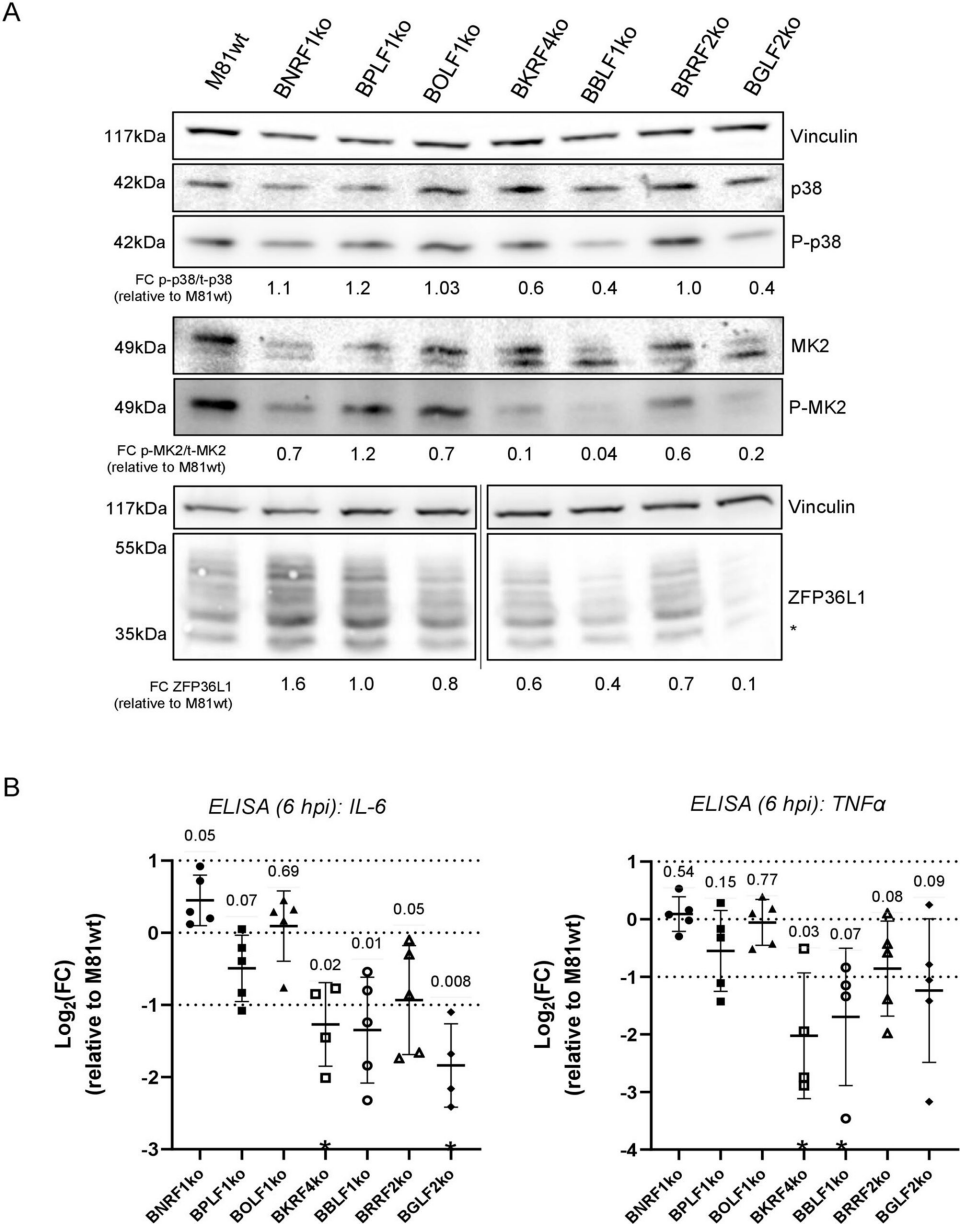
EBV tegument proteins induce p38/MK2 activation and ZFP36L1 expression. (**A**) Primary CD19^+^ B cells were infected for 6 hours with different tegument protein knockouts and immunoblotted to detect p38 and MK2 activation and ZFP36L1 expression. Vinculin was used as a loading control. *n* = 5 independent biological replicates. A representative fold change versus the M81wt control is reported for the total to phosphorylated ratios for p38 and MK2, as well as for the quantified ZFP36L1. We detected p38 and MK2 expression in the set of samples also used for the experiments described in Fig. 2G. Thus, Fig. 6A and 2G share the same vinculin loading control. (**B**) The conditioned medium from (**A**) was used to quantify via ELISA the secretion of IL-6 and TNFα. Values are expressed as Log_2_(fold change) relative to M81wt-infected cell. The value for each single replicate is shown, and the mean ± SD is reported (*n* = 5 independent biological replicates). An asterisk on the *x*-axis is used to indicate when samples were below the detection limit and could not be quantified. Statistical analysis was performed using a one-sample *t*-test (*μ* = 0). *P* values are reported above the comparison. *P* ≤ 0.05 was considered statistically significant.

Because EBV VLPs can induce p38-MK2 activation and ZFP36L1 transcription and translation in the absence of viral DNA, we concluded that components of the VLPs are responsible for this effect. Therefore, we infected B cells with our tegument knockout panel. We found that, in the absence of BBLF1 or BGLF2, and to a lesser extent BKRF4, p38/MK2 and ZFP36L1 failed to be activated upon infection (Fig. 6A). Quantification of cytokine release after infection with these defective mutants showed a similar pattern. In the absence of BBLF1, BGLF2, or BKRF4, TNFα and IL-6 release were markedly reduced (Fig. 6B). Unexpectedly, infection with the ∆BNRF1 deletion mutant gave rise to cytokine release levels comparable to or even higher than after infection with wild-type EBV (Fig. 6B).

### STAT3 and p38/MK2 signaling allow early latent gene expression

**Fig 7:**
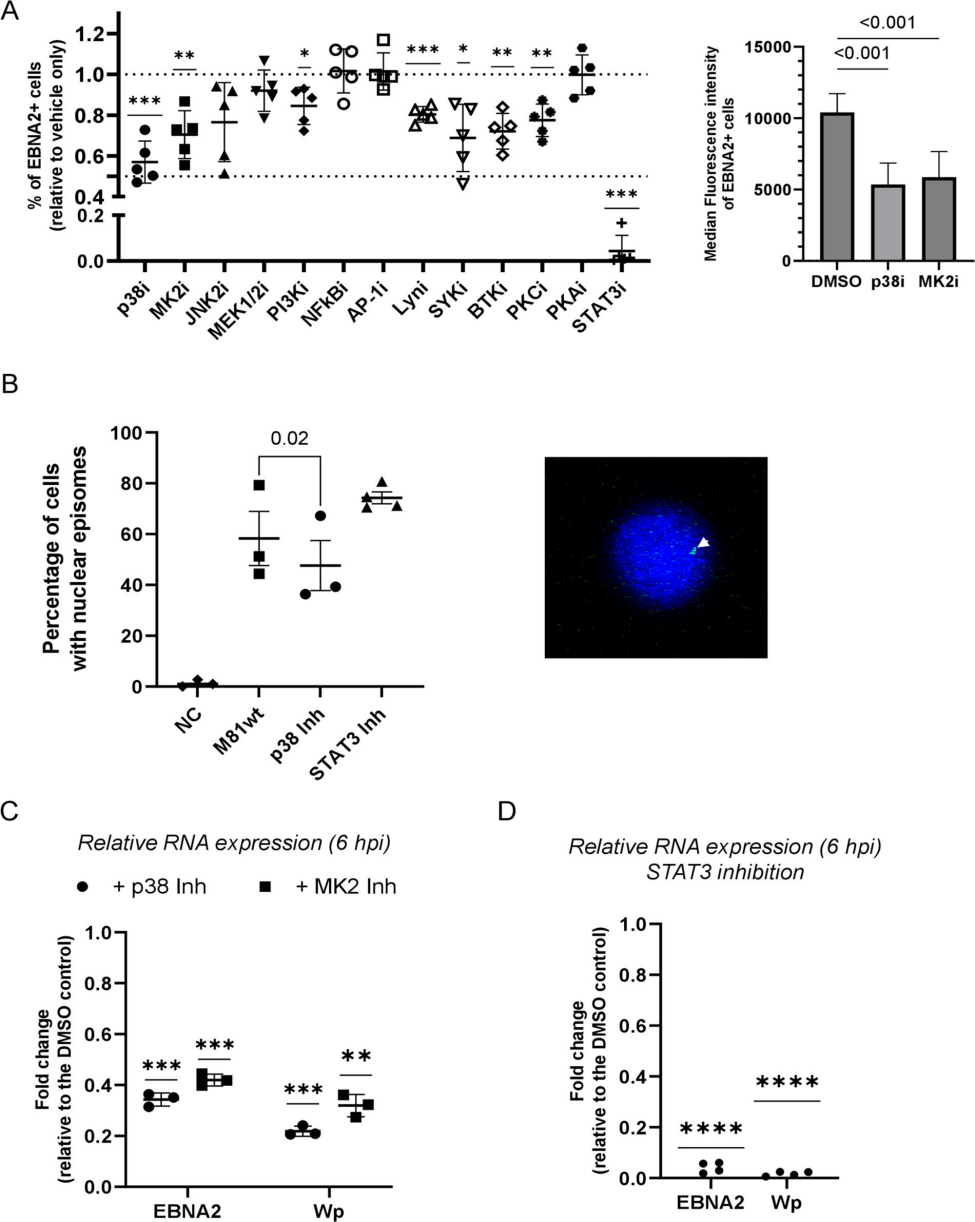
p38 /MK2 activation allows viral DNA transport and early latent gene expression. (**A and B**) The role played by several signaling pathways in the establishment of EBV infection was evaluated using selective pathway inhibitors. Flow cytometry analysis was performed at 16 hours post-infection to determine the percentage of EBNA2-positive cells (**A**). The percentage of EBNA2-positive cells is reported, with each single replicate being shown, as well as the mean ± SD (*n* = 5 independent biological replicates) (left). The median fluorescence intensity for EBNA2 in the EBNA2+ cells from the left panel is reported for M81wt-infected cells in the presence of vehicle only (DMSO), p38 inhibitor, and MK2 inhibitor (right). A one-sample *t*-test (*μ* = 1) (left) or a one-way ANOVA with Dunnett’s post hoc test (right) were performed for statistical analysis. (**B**) FISH was used to detect EBV episomal DNA in freshly M81wt-infected CD19^+^ B cells in the presence or absence of the p38 inhibitor or of the STAT3 inhibitor (3 hours post-infection). The percentage of cells showing nuclear episomes is reported, with each single replicate being shown, as well as the mean ± SD. Three (p38 inhibitor-treated) or 4 (STAT3 inhibitor-treated) independent biological replicates were analyzed. A representative image of an EBV nuclear episome is shown (right panel, white arrow). The EBV episomal DNA is shown in green, while the nucleus was counterstained with DAPI (blue). (**C and D**) mRNA expression levels for EBNA2 and Wp promoter-originated transcripts were quantified by RT-qPCR in CD19^+^ B cells exposed to M81wt virus in the presence of either the p38 inhibitor, the MK2 inhibitor (**C**), or STAT3 inhibitor (**D**) at 6 hours post-infection. The fold change in the expression levels between the treated sample and the untreated sample is reported for each replicate, and the mean ± SD is also shown. *n* = 3 (**C**) or 4 (**D**) independent biological replicates. (**B–D**) Statistical analysis was performed using a paired two-tailed *t*-test (**B**) or a one-sample *t*-test (*μ* = 0) (**C and D**). *P* values are reported above the comparison or indicated with a star notation (*P ≤ 0.05, **P ≤ 0.01, ***P ≤ 0.001, and ****P ≤ 0.0001). *P* ≤ 0.05 was considered statistically significant.

We then set out to determine whether the signaling pathways activated after EBV infection have an impact on the establishment of active latency. To this end, we treated B cells exposed to wild-type virus with an extended panel of inhibitors and assessed EBV infection by detection of the viral protein EBNA2 at 16 hours post-infection by flow cytometry (Fig. 7A). In parallel, drug toxicity was evaluated using the alamarBlue viability reagent (Fig. S7A). Inhibition of the p38-MK2 axis or of STAT3, and to a lesser extent of the BCR-associated kinases, showed the strongest reduction in the number of latently infected B cells (Fig. 7A). Interestingly, treatment with inhibitors of either p38 or MK2 not only reduced the percentage of EBNA2-positive cells but also halved EBNA2 expression levels in infected cells (Fig. 7A). Only the STAT3 inhibitor Stattic showed significant cell toxicity at this time point, although the reduced viability was not sufficient to justify the almost complete absence of EBNA2+ cells upon treatment with the inhibitor (Fig. S7A). To identify the steps of the infection process that are modulated by these kinases, we performed fluorescent *in situ* hybridization (FISH) with a probe specific to the viral genome (Fig. 7B). This assay showed that inhibition of the p38 pathway led to a 25% reduction in the number of cells carrying viral episomes in their nucleus. A similar analysis using B cells exposed to ∆BGLF2 knockout revealed a much stronger effect with a 75% reduction in cells carrying the viral genome, suggesting that the functions of this tegument protein extend further than just p38 activation (Fig. S7B). Recent work has, for example, shown that BGLF2 has a profound effect on miRNA expression (73). However, STAT3 inhibition did not impede transport and injection of the viral DNA into the nucleus of infected cells, confirming the limited toxicity of the inhibitor (Fig. 7B). Finally, we evaluated the impact of p38 and STAT3 inhibition on the expression of latent gene transcripts at 6 hours post-infection (Fig. 7C and D). At this time point, EBNA2 expression was close to the levels seen in established LCLs and transcription of other EBNA family members had already started (Fig. S7C). Blocking of p38 or MK2 activation led to a strong reduction in the expression of both EBNA2 and Wp promoter-driven transcripts. This effect was stronger after p38 inhibition than after MK2 inhibition, suggesting a possible broader activation of the p38 signaling cascade than the sole engagement of the MK2 branch. STAT3 inhibition reduced Wp-driven transcription and EBNA2 transcription even more potently.

## DISCUSSION

**Fig 8:**
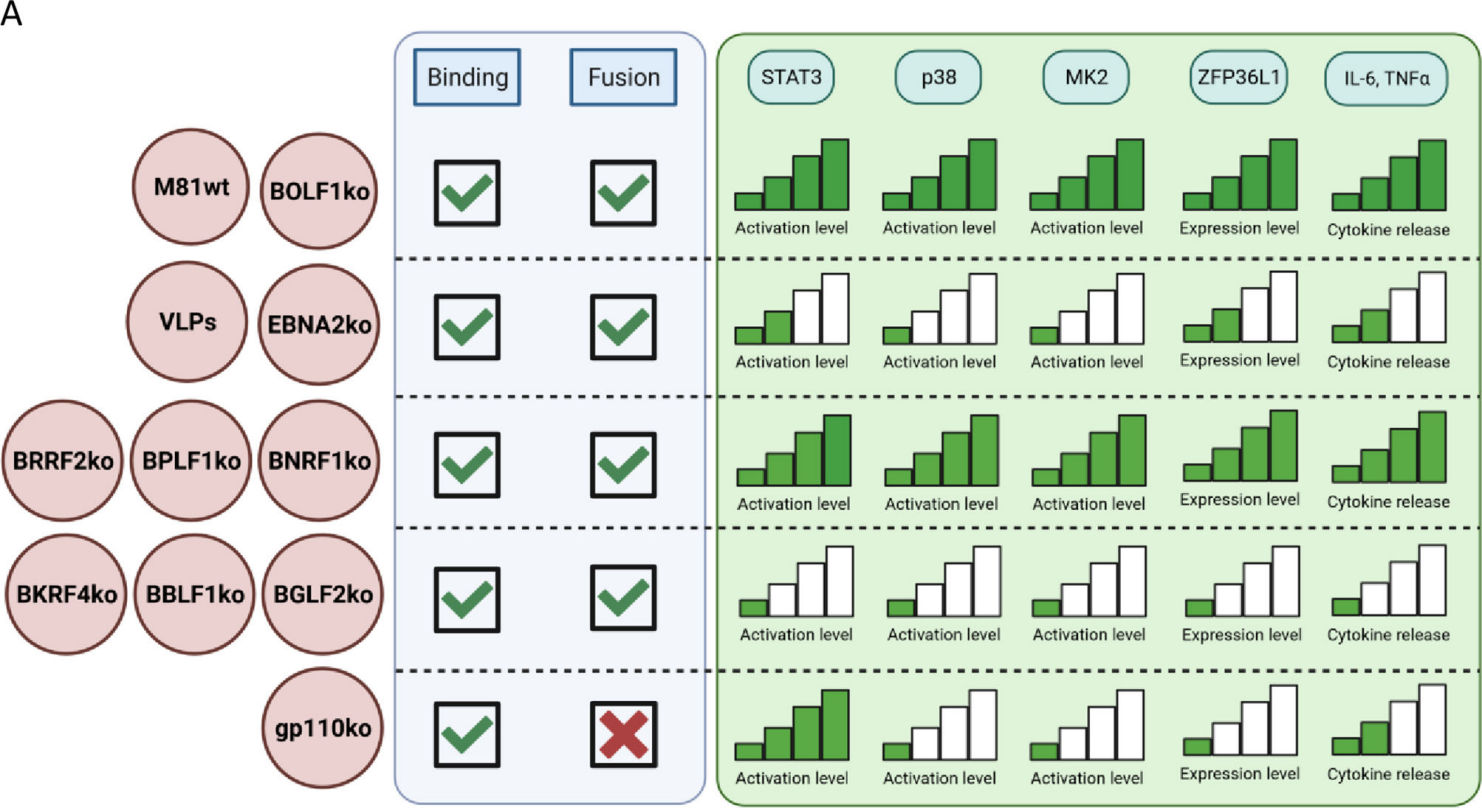
Graphical summary of the results. The induction levels for each of the target investigated are shown relative to the recombinant virus used (one step 25%, two steps 50%, three steps 75%, and four steps 100% of wild-type activity), together with their ability to bind and to fuse with target cells. Image created with BioRender.com.

This paper reports that the STAT3 and p38-MK2-ZFP36L1 pathways are active early after EBV infection. BCR-associated kinases, in particular Syk, but also PKA and NF-κB modulated this process. Activation of both STAT3 and p38 was essential for the initiation of viral active latency and caused a short-lived spike in cytokine release. Indeed, proinflammatory signals elicited by STAT3 and p38 were limited by ZFP36L1 expression. However, STAT3 and p38 did not, or only mildly, affect viral DNA transport and its injection into the nucleus. Viral mutants lacking a surface glycoprotein or a tegument protein were instrumental to ascribe full STAT3 activation to virus binding and to active latency (Fig. 8). Because STAT3 is less active in viruses that can enter the cytoplasm but cannot initiate latency, it is likely that post-binding events first downregulated this pathway before latency upregulated it again. p38-MK2-ZFP36L1 activation followed intra-cytoplasmic entry of the virus and latency activation. However, VLPs activated the different members of the p38-MK2-ZFP36L1 pathway with substantial, though reduced efficacy (between a third and a half), relative to wild-type viruses. VLPs and the M81/∆EBNA2 behaved similarly, but it is interesting to note that the former was consistently slightly more efficient at activating the different members of the p38-MK2-ZFP36L1 pathway. This suggests that recognition of the viral DNA dampens this genetic cascade.

The observation that the viral particle provides an activation signal that initiates active latency solves a potential paradox. Indeed, the Wp promoter needs first to be activated to transcribe EBNA2 and initiate active latency. EBNA2 in turn can activate a second promoter, Cp, located upstream of Wp that itself activates transcription of all EBNA genes (14, 74 – 76). However, this self-reinforcing loop is not functional for Wp that relies on cellular transcription factors for its firing. BSAP/PAX5 has previously been identified as such a transactivator. We now report that activating signals coming from the particle contribute to Wp activation. STAT3 activation has previously been proposed to allow freshly infected B cells to progress through the cell cycle and to block apoptosis (16 – 18). The present study defines the activation of latent gene expression as a new function for STAT3 early after infection. Interestingly, STAT3 activated latent gene expression, but latent protein expression also boosted p-STAT3 levels. This suggests the existence of another reinforcing loop between both events.

The STAT3 and p38 pathways control the inflammatory response, especially in immune cells (77 – 79). Indeed, we found that their inactivation by selective inhibitors dampened cytokine release after B cell infection. Viral mutants that can bind but cannot fuse with their target cells also show that two distinct events are responsible for cytokine release after EBV infection. Virus binding itself led to limited cytokine transcription and release, as previously reported (27). However, latent gene expression and, to some extent, the presence of viral DNA in the infectious particle, induced a further boost in cytokine transcription that resulted in larger cytokine secretion. Nevertheless, TNFα and IL-6 release after EBV infection was inefficient in comparison with other stimuli, such as TPA or LPS. Moreover, it was limited to the first 24 hours post-infection and IL-6 and TNFα transcripts decreased in abundance rapidly after an initial burst 2 hours post-infection, suggesting that cytokine release is actively repressed in infected cells. This could be ascribed to STAT3 and p38’s abilities to induce the simultaneous expression and phosphorylation of ZFP36L1, an RNA-binding protein that negatively regulates cytokine transcription. Indeed, ZFP36L1’s transcription profile over time was exactly opposite to those of the cytokines. Moreover, a knock-down experiment using a specific siRNA showed that downregulation of ZFP36L1 during the first 6 hours of infection led to an increase in IL-6 transcription. Thus, EBV particles can activate latent gene expression without causing a strong and prolonged cytokine response.

Using MK2 and p38 inhibitors, we found that the p38-MK2 axis controls ZFP36L1 induction. This observation is in line with previous work showing that MK2 both activates ZFP36 family protein transcription and phosphorylates the resulting ZFP36 proteins (63 – 65). The phosphoproteomic analysis recorded the presence of phospho-events for both the p38 pathway and ZFP36L1 after virus binding. However, the intensity of activation was minimal in comparison to downstream events such as virus entry and latent protein production. ZFP36L1 can be phosphorylated at multiple sites that can be either activating (56) or inactivating (52, 53, 80). ZFP36L1 phosphorylation allows direct interaction with the chaperone 14-3-3 and thereby its stabilization. However, these events also inactivate the ability of the protein to promote the degradation of its RNA targets. Although MK2 is considered as the main regulator of ZFP36L1 phosphorylation, other kinases such as PKA (56) and PKB (52, 80) have been shown to target this RNA-binding protein. We found here that SYK, LYN, BTK, and STAT3 can also directly or indirectly influence ZFP36L1 expression and its phosphorylation status in B cells.

Modulation of the p38/MK2 pathway has been previously reported after infection with other members of the *Herpesviridae* family. However, these events occurred in already-infected cells and were thus independent of the virion (81, 82). For example, infection with the Kaposi Sarcoma Herpesvirus (KSHV) induced the p38-MK2 pathway via expression of the Kaposin B protein (83). Phosphorylated MK2 caused the activation of the MK2-HSP27-p115RhoGEF-RhoA signaling axis which, in turn, disrupted the P-body network, a set of cytoplasmic hubs involved in the processing of mRNAs targeted for degradation (84, 85). In contrast to EBV, KSHV-infected cells produce large amounts of cytokines that are beneficial to cell growth. Thus, there is no need to block cytokine release following MK2 activation (86). Interestingly, HCMV infection does not induce but rather represses ZFP36 expression to maintain high levels of IL-10 transcription and secretion. This cytokine dampens a potential T cell response against infected cells (87).

Early studies performed using either recombinant soluble gp350 or wild-type EBV showed that IL-6 secretion upon binding was dependent on the NF-κB p65/p50 and NF-IL-6 transcription factors (27). However, we could not identify an increase in the level of phosphorylated p65 after the infection of primary B cells. The results obtained using the selective NF-κB inhibitor PS-1145 clearly showed a very limited effect on IL-6 and TNFα secretion. This fits with reports that describe BPLF1 and BGLF2, as well as the gp110 glycoprotein as active repressors of the NF-κB pathway during the early phases of infection (28 – 31).

We then used a panel of 12 viral mutants to identify the precise steps of the infection process at which these molecular events took place (Fig. 8). We found that STAT3 phosphorylation occurred after virus binding and did not require any of the EBV fusion proteins. However, it required Syk for full activation. More work will be necessary to understand how EBV binding to B cells activates STAT3 at the molecular level. However, we also found evidence of STAT3 modulation at the post-fusion step of infection. Virions that lack BGLF2, BBLF1, or BKRF4 showed low STAT3 levels. Because this set of mutants also failed to activate p38/MK2, it is possible that their effect on STAT3 results from their stimulating effects on p38/MK2 that itself potentiates STAT3 activation (88, 89). In contrast, mutants that lack BNRF1 or BPLF1 induced p-STAT3 and p-p38/MK2 expression at levels higher than seen with EBV VLPs. Altogether, this suggests that different tegument proteins can modulate STAT3 and p38/MK2 activation negatively and positively, perhaps at different stages of infection. A link between p38 and BGLF2 has previously been identified during viral lytic replication (40, 90, 91). Our observations suggest that this interaction is also important at the initial phase of virus-cell interactions. Because BBLF1 acts as a chaperone for BGLF2 (92), it is likely that the failure in p38 induction observed in its absence is due to its action on BGLF2. Although BOLF1, another tegument protein, was speculated to interact indirectly with BGLF2 via BKRF4, we found here that a BOLF1 null virus fully activated p38 and ZFP36L1 (93). The phenotype of viruses lacking one tegument proteins is generally mild in terms of replication and egress (92 – 99). This contrasts with our observation that members of the BGLF2 complex play a crucial role in infection.

The data described in this paper are reminiscent of the ability of Alpha- and Betaherpesviruses to package transactivators such as VP16 and pp71 in their tegument to directly activate transcription of viral Immediate Early (IE) genes upon release, but this direct mechanism has apparently not been conserved for EBV (100, 101). Although BNRF1 shares the ability of VP16 to disrupt the ATRX-Daxx interaction and BPLF1 is a sequence homolog of pp71, we did not find any evidence that these two EBV proteins directly activate viral transcription (102, 103).

In summary, this work shows that the role of viral particles in EBV infection is not restricted to the successful transfer of the viral DNA into infected cells but extends to the inception of the latent phase of infection and to the control of the immune response.

## MATERIALS AND METHODS

### Experimental model

#### Cell lines and primary cells

The HEK293 cell line is a neuro-endocrine cell line obtained by transformation of embryonic epithelial kidney cells with adenovirus (ATCC: CRL-1573). Peripheral blood mononuclear cells from buffy coats purchased from the blood bank of the University of Heidelberg (IKTZ) were purified on a Ficoll cushion, and CD19^+^ primary B-lymphocytes were isolated using Dynabeads CD19 Pan B (Invitrogen), and beads were detached using DETACHaBEAD CD19 (Invitrogen).

Cells were routinely cultured in RPMI-1640 medium (Invitrogen) supplemented with 10% fetal bovine serum (FBS) (Merck). Primary B cells were cultured with RPMI-1640 supplemented with 20% FBS + 1% HEPES. All cells were cultured at 37°C with 5% CO2.

### Reagents

#### Pathway inhibitors

Stock solutions of pathway inhibitors were prepared in either DMSO or water according to the manufacturer’s instructions. We used inhibitors which targeted selectively the following pathways: p38 (SB239063, 10 µM), MK2 (PF-3644022, 10 µM), JNK (SP600125, 10 µM), MEK1/2 (trametinib, 0.1 µM), PI3K (ZSTK474, 1 µM), NF-κB (PS-1145, 3 µM), AP-1 (T-5224, 20 µM), Syk (PRT-062607, 2 µM), Lyn (bafetinib, 5 µM), BTK (ibrutinib, 10 nM), PKC (bisindolylmaleimide I, 5 µM), PKA (H-89, 5 µM), STAT3 (Stattic, 2.5 µM), and JAK2 (AG490, 25 µM). In addition, actinomycin D (2 µg/mL) and cycloheximide (15 µg/mL) were used to inhibit transcription and translation, respectively.

#### Antibodies

We stained infected cells with mouse monoclonal antibodies against EBNA2 (clone PE2) and the Alexa Fluor 488-conjugated goat anti-mouse secondary antibody (Invitrogen). ZFP36L1 staining was performed with a rabbit monoclonal antibody (BRF1/2, Cell Signaling Technology #2119, 1:200) and a Cy-3-conjugated goat anti-rabbit secondary antibody (Dianova, Invitrogen). We performed western blots with mouse monoclonal antibodies against IκBα (BioLegend, # 609101, 1:1,000), vinculin (Santa Cruz Biotechnology, sc-73614 1:1,000), actin (Santa Cruz Biotechnology, sc-8432, 1:1,000), phospho-ATF2 Thr71 (Santa Cruz Biotechnology, sc-8398, 1:200), and MK2 (Santa Cruz Biotechnology, sc-393609, 1:500). We used rabbit monoclonal antibodies from Cell Signaling Technology against phospho-ERK Thr202/Tyr204 (#4370, 1:1,000), phospho-p65 (#3033, 1:1,000), phospho-STAT3 Tyr705 (#9131, 1:1,000), STAT3 (#4904, 1:1,000), phospho-p38 Thr180/Tyr182 (#9211, 1:1,000), p38 (#9212, 1:1,000), phospho-MK2 Thr334 (#3007, 1:1,000), and BRF1/2 (#2119, 1:1,000). A rabbit monoclonal antibody against phospho-AKT1 Ser473 from Abcam was used (#ab81283, 1:10,000). The anti-phospho MSK2 Ser196 was a rabbit polyclonal antibody purchased from R&D (#AF189, 1:500).

### Construction of recombinant viruses and virus production

The wild-type EBV strain M81 is available as a recombinant BACMID (104). The viral genomes were cloned onto a prokaryotic F-plasmid that carries the chloramphenicol (Cam) resistance gene, the gene for green fluorescent protein (GFP), and the hygromycin resistance gene (B110). All PCR primers used for PCR cloning or En Passant mutagenesis are listed in Table S2.

For the generation of the BOLF1 (B1636), BBLF1 (B1651), BRRF2 (B1669), and BGLF2 (B1672) knockouts, we used En Passant mutagenesis. For the generation of the BZLF2 (B279), BXLF2 (B1789), BALF4 (B1001), BPLF1 (B1580), and BKRF4 (B1641) knockouts, we used recombination. BNRF1 (B1099) and EBNA2 (B975) knockouts were generated by recombination with flp sites to later remove the selection cassette introduced. More details on how the viral recombinants were generated are available in Table S3. The BNRF1 and VLPs recombinants were previously described (105).

### Oligonucleotides and probes

All synthesized oligonucleotides and probes used in this study are listed in Table S2.

### Stable transfection of EBV-BAC and plasmid rescue into *Escherichia coli*

Recombinant EBV plasmids were lipotransfected into HEK293 cells using Metafectene (Metafectene, Biontex), and the selection of stable HEK293 cell clones carrying the recombinant EBV plasmid was achieved by adding hygromycin to the culture medium (100 µg mL^−1^) as previously described (106). To assess the genome integrity of recombinant EBV within the stable clones, the circular EBV genomes present in these cells were extracted using a denaturation-renaturation method and transferred into the *E. coli* strain DH10B by electroporation (1,200 V, 25 µF, and 200 Ω). The transformed *E. coli* clones were further assessed by restriction-enzyme analysis of plasmid minipreps.

### Virus induction

The HEK293 cells stably carrying the recombinant EBV BACs were transfected with expression plasmids encoding BZLF1 (p509) and BALF4 (pRA) using the liposome-based transfectant Metafectene (Biontex). Three days after transfection, viral supernatants were collected and filtered through a 0.45-µm filter. For the BALF4 knockout producer cell line, the induction was performed without the BALF4-encoding plasmid. VLP production was performed following the same protocol as for the other EBV BAC-carrying cell lines by transfecting both the BZLF1 (p509) and BALF4 (pRA) plasmids.

### Quantification of viral titers

To evaluate EBV genome equivalents per milliliter of supernatant, viral supernatants were treated with DNase I. Following a subsequent treatment with proteinase K, we used qPCR with primers and probes specific for the non-repetitive EBV BALF5 gene sequence to measure the EBV copy numbers in the supernatants (94).

### Quantification of gp350+ particles

M81 wild-type virus, previously quantified with reverse transcription qPCR, was titrated (1, 0.75, 0.5, and 0.25 × 10^7^ genome-containing particles) and bound to human primary B cells at 4°C. Cells were washed, stained with α-gp350 (clone 72A1) and α-mouse IgG-Cy3 antibodies, and analyzed with flow cytometry. Median fluorescence intensity (MFI) values were determined for different amounts of virus. A standard curve was generated for EBV genomes versus MFI. This assay revealed the number of gp350+ infectious particles per milliliter of supernatants. Concurrently, supernatants containing either VLPs or various knockout viruses were incubated with human primary B cells and stained as above. MFI values obtained for VLPs or various knockout viruses were extrapolated off the standard curve to quantify the concentration of these particles (107).

### B cell infections

Purified CD19+ human B cells were exposed to viral supernatant with a multiplicity of infection of 30 by rolling for two hours at +4°C. The MOI was calculated based on qPCR viral titer quantification or gp350+ particle quantification. The latter was used if different virus recombinants were compared in the same experiment. Cells were then cultured at a density of 2 × 10^6^ cell/mL for the indicated amount of time with RPMI-1640 supplemented with 20% FBS + 1% HEPES at 37°C. For the experiments in which pathway inhibitors were used, these were added to primary B cells at the concentration indicated for 1 hour prior to the incubation with a virus supernatant. The inhibitors were then added at each of the following steps maintaining the same final concentration. Cell viability was monitored during treatment with the inhibitors using the alamarBlue viability reagent (Invitrogen, DAL1025) according to the manufacturer’s instructions. Fluorescence was measured using a plate reader (Ex 560 nm, Em 590 nm). Neutralization experiments with the AMMO1 antibody were performed by pre-incubating the virus with the AMMO1 antibody at a concentration of 5 µg/mL for 1 hour at room temperature while rolling. The antibody was added at the same concentration once the cells were incubated with the virus and during each subsequent step of the experiment.

Treatment of B cells with the M81 virus-conditioned medium was performed after two rounds of filtration of M81wt virus supernatant with a 0.22-µm filter.

### ZFP36L1 knock-down

Purified CD19+ human B cells were cultured for 3 days in the presence of 100 ng/mL soluble recombinant human CD40L and 20 ng/mL of recombinant human IL-4 at a concentration of 2 × 10^6^. After 3 days, cells were counted, washed once with PBS, and resuspended in P3 Primary electroporation buffer (Lonza, V4SP-3096) with supplements according to the manufacturer’s instructions. For each condition, 2 × 10^6^ cells were electroporated in 20 µL in the presence of 300 pmol of Silencer Select Negative Control No. 1 siRNA (Invitrogen, 4390843) or Silencer Select s2091 (Invitrogen, 4392420). To each reaction, an equimolar amount of a siRNA transfection indicator was used to assess electroporation efficiency (Horizon Discovery, D-001630–02-05). Electroporation was performed using the 4D-Nucleofector X Unit (Lonza, AAF-1003X) and the protocol DN107. Cells were transferred to pre-warmed medium, and 4-hour post-electroporation cells were stimulated with shCD40L and rhIL-4 at the same concentration. Two-day post-electroporation cells were infected with the M81wt virus for 6 hours, after which they were processed for RNA extraction.

### Proteomic and phosphoproteomic analysis

#### Proteomic analysis

##### Sample preparation

Proteins (10 μg) were separated for 0.5 cm via a SDS-PAGE. After Commassie staining, the total sample was cut out and used for subsequent Trypsin digestion according to a slightly modified protocol described by Shevchenko et al. (108) on a DigestPro MSi robotic system (INTAVIS Bioanalytical Instruments AG).

##### Mass spectrometry run

Liquid-chromatography-mass spectrometry (LC-MS/MS) analysis was performed using an Ultimate 3000 ultra-performance liquid chromatography (UPLC) system connected to a Q-Exactive HF-X mass spectrometer. The UPLC was operated using a trap-elute setup. Peptides were first loaded on a trap column (Acclaim PepMap300 C18, 5 µm, 300 Å wide pore; Thermo Fisher Scientific) for 5 minutes and 30 µL/minute of 0.05% trifluoroacetic acid (TFA) in water. During the analytical gradient (solvent A: water with 0.1% formic acid; solvent B: 80% acetonitrile, 20% and 0.1% formic acid) the concentration of solvent B was ramped from 2% to 25% (150 min) and 25%−40% (30 minutes) on a nanoEase MZ Peptide analytical column (300 Å, 1.7 µm, 75 µm × 200 mm; Waters). Eluting peptides were analyzed by a Q-Exactive-HF-X mass spectrometer (Thermo Fisher Scientific) running in data-dependent acquisition mode. A full scan at 120 k resolution was followed by up to 35 MS/MS scans at 15 k resolution. Precursors were isolated for MS/MS scans via a quadrupole isolation window of 1.6 m/z and fragmented via a collision energy of 27 NCE. Unassigned and singly charged peptides were excluded from fragmentation, and dynamic exclusion was set to 60 seconds.

##### Data analysis

Data analysis was performed by MaxQuant (109) (version 1.6.0.16) using an organism-specific database extracted from Uniprot.org under default settings. Identification False Discovery Rate (FDR) cut-offs were 0.01 on the peptide level and 0.01 on the protein level. The match between runs option was enabled to transfer peptide identifications across Raw files based on accurate retention time and m/z.

Quantification was done using a label-free quantification approach based on the MaxLFQ algorithm (110). A minimum of two quantified peptides per protein was required for protein quantification.

Data have been further processed by in-house-compiled R-scripts to plot and filter data.

##### Statistics

The Perseus software package (version 1.6.7.0) using default settings for further statistical analysis (111) of LFQ data (https://yeroslaviz.github.io/coxdocs.2023/perseus_instructions.html) was used.

### Phosphoproteomic analysis

#### Sample preparation

4 × 10^6^ primary B cells from healthy human donors were left uninfected or infected with M81wt, VLPs, or gp110 knockout virus for 6 hours. A total of 21 donors were used, and the same amount of protein was pooled from seven donors for each condition to generate three independent pooled biological replicates. Samples have been prepared according to a slightly modified protocol described by Potel et al. (112). Briefly, cell pellets were resuspended with lysis buffer (100 mM Tris-HCl pH 8.5, 7 M urea, 1% Triton, 10 U/mL DNase I, 1 mM magnesium chloride, 1% benzonase, 1 mM sodium orthovanadate, phosphoSTOP phosphatase inhibitors, and complete mini-EDTA-free protease inhibitors) and lysed by sonication. Cell debris was removed by ultracentrifugation (140,000 × *g* for 1 hour at 4°C). One percent benzonase was added to the supernatant followed by incubation at room temperature for 2 hours. Protein concentration was determined and followed by chloroform/methanol precipitation as described by Wessel et al. (113).

Pellets were resuspended in digestion buffer (8 M urea, 100 mM NaCl, 50 mM triethylammonium bicarbonate [TEAB], pH 8.5), followed by reduction in 10 mM dithiothreitol (DTT) for 1 hour at 27°C, alkylation by 30 mM iodoacetamide for 30 minutes at room temperature in the dark, and quenching the reaction by adding an additional 10 mM DTT.

Samples have subsequently been digested by Lys-C at an enzyme:protein ratio of 1:00 for 3–4 hours at 30°C, diluted with 50 mM TEAB to a resulting urea concentration of 1.6 M and further digestion with trypsin overnight at 37°C in an enzyme:protein ratio of 1:50. Digestion was stopped by acidification, adding 0.02% (vol/vol) TFA.

Digested peptides have been desalted using C18 SepPack Cardridges and resuspended in 0.07% (vol/vol) TFA in 30% (vol/vol) acetonitrile (ACN) and fractionated by on-column Fe^3+^-immobilized metal affinity chromatography (IMAC) enrichment on an Ultimate 300 LC system using the method described by Ruprecht et al. (114). The two fractions per sample, containing mainly either unphosphorylated or phosphorylated peptides, have been desalted by StageTips (115) and resolved in 50 mM citric acid and 0.1% TFA.

#### Mass spectrometry run

LC-MS/MS analysis was performed using an Ultimate 3000 UPLC system connected to a Q-Exactive HF-X mass spectrometer. The UPLC was operated using a trap-elute setup. Peptides were first loaded on a trap column (Acclaim PepMap300 C18, 5 µm, 300 Å wide pore; Thermo Fisher Scientific) for 5 minutes and 30 μL/min of 0.05% TFA in water. During the analytical gradient (solvent A: water with 0.1% formic acid; solvent B: 80% acetonitrile, 20% and 0.1% formic acid) for the phospho-fraction, the concentration of solvent B was ramped from 2% to 8% (15 min), 8%−25% (135 min), and 25%–40% (20 min), and for the full proteome samples, the concentration of solvent B was ramped from 2% to 25% (150 min) and 25%−40% (30 min). For separation, a nanoEase MZ Peptide analytical column (300 Å, 1.7 µm, 75 µm × 200 mm; Waters) was used. Eluting peptides were analyzed by a Q-Exactive-HF-X mass spectrometer (Thermo Fisher Scientific) running in data-dependent acquisition mode. A full scan at 120 k resolution was followed by up to 35 MS/MS scans at 15 k resolution. Precursors were isolated for MS/MS scans via a quadrupole isolation window of 1.6 m/z and fragmented via a collision energy of 27 NCE. Unassigned and singly charged peptides were excluded from fragmentation, and dynamic exclusion was set to 60 seconds.

#### Data analysis

Data analysis was carried out by MaxQuant (109) using an organism-specific database extracted from Uniprot.org under default settings. Identification FDR cut-offs were 0.01 on the peptide level and 0.01 on the protein level. The match between runs option was enabled to transfer peptide identifications across Raw files based on accurate retention time and m/z.

Quantification was done using a label-free quantification approach based on the MaxLFQ algorithm (110). A minimum of two quantified peptides per protein was required for protein quantification.

Data have been further processed by in-house-compiled R-scripts to plot and filter data.

#### Statistics

The Perseus software package (version 1.6.7.0) using default settings for further statistical analysis (111) of LFQ data (https://yeroslaviz.github.io/coxdocs.2023/perseus_instructions.html) and phospho data (https://yeroslaviz.github.io/coxdocs.2023/perseus_instructions.html) was used. Adapted from the Perseus recommendations (111), protein groups with non-zero intensity values in 70% of the samples of at least one of the conditions were used and imputation with random values drawn from a downshifted (1.8 standard deviation) and narrowed (0.3 standard deviation) intensity distribution of the individual sample. Pathway analysis for the identified phosphopeptides was performed using the QIAGEN IPA (QIAGEN Inc., https://digitalinsights.qiagen.com/IPA) software (116).

### Western blot analysis

Proteins were extracted with a standard lysis buffer (150 mM NaCl, 0.5% NP-40, 1% sodium deoxycholate, 0.1% SDS, 5 mM EDTA, 20 mM Tris-HCl pH 7.5) and 1× protease and phosphatase inhibitor cocktail (Halt Protease and Phosphatase Inhibitor, Thermo Scientific) for 15 minutes on ice followed by sonication to shear the genomic DNA. Between 20 and 30 µg of proteins was denatured in Laemmli buffer for 10 minutes at 95°C, separated on 12.5% SDS–polyacrylamide gels, and electroblotted onto a 0.22-µm nitrocellulose membrane (Hybond C, Amersham). A blocking step was performed for 1 hour at room temperature by incubating the blots in TBS with 0.1% Tween 20 and 5% milk dry, after which the antibody against the target protein was added and the blot was incubated overnight at 4°C. To allow simultaneous detection of multiple proteins of interest, nitrocellulose membranes were cut and the resulting membrane sections separately exposed to antibodies. Accordingly, these immunoblots share the same loading control (vinculin). Antibodies recognizing phosphorylated proteins were incubated in tris-buffered saline(TBS) with 0.1% Tween 20 and 5% bovine serum albumin (BSA). After extensive washing in TBS with 0.1% Tween 20, the blot was incubated for 1 hour at room temperature with suitable secondary antibodies coupled to horseradish peroxidase [goat anti-mouse (Promega) or goat anti-rabbit (Promega) lgG]. Bound antibodies were detected using the enhanced chemiluminescence (ECL) detection reagent (Pierce). Quantification of the acquired signal was performed on 16-bit TIF files using the Fiji software (117).

### Subcellular fractionation

The subcellular fractionation protocol was modified from a previously published method (118). In summary, 6 × 10^6^ uninfected or M81wt-infected primary CD19^+^ B cells were washed once with PBS 1× and gently resuspended in 40 µL of buffer A (10 mM HEPES pH 7.9, 10 mM KCl, 0.1 mM EDTA, 1 mM DTT, 1× protease and phosphatase inhibitor cocktail). Cells were then incubated on ice for 15 minutes, and then 2.5 µL of 10% NP-40 was added before being vortexed at minimum speed for 10 seconds and incubated on ice for 2 minutes. The supernatant was collected after centrifuging the samples for 6 minutes at 600 *× g* at 4°C and identified as the cytoplasmic fraction. The pellet was then gently washed once with 100 µL of buffer A, centrifuged for 5 minutes at 600 × *g* at 4°C, and resuspended in 15 µL of buffer B (20 mM HEPES pH 7.9, 400 mM NaCl, 1 mM EDTA, 1 mM DTT, 1× protease and phosphatase inhibitor cocktail). Resuspended pellets were then vortexed at maximum speed for 15 seconds, incubated on ice for 15 minutes, and vortexed once more prior to proceeding to sonication. Samples were then centrifuged at maximum speed for 5 minutes at 4°C, and the supernatant was identified as the nuclear fraction. Both the cytoplasmic and the nuclear fractions were quantified and further processed for western blot.

### Lambda phosphatase treatment

Total protein lysate prepared with EDTA-free RIPA buffer and protease and phosphatase inhibitors was treated for 30 minutes at 30°C with lambda protein phosphatase according to the manufacturer’s instructions (New England Biolabs). An untreated control was processed similarly in the absence of the enzyme. Samples were then analyzed by western blot.

### Flow cytometry

Cells were resuspended in 100 µL of PBS and fixed by adding 100 µL of 4% paraformaldehyde in PBS. After 20 minutes at room temperature, cells were washed and permeabilized using 100% methanol for 10 minutes at −20°C. After permeabilization, cells were washed once and resuspended in 100 µL. The staining was performed by incubating the primary antibody diluted in PBS + 2% FBS for 1 hour at room temperature. After washing, cells were resuspended in 100 µL of secondary antibody conjugated to Cy-3 diluted in PBS + 2% FBS. Staining was performed for 1 hour at room temperature. An unstained control, an isotype control, and a secondary antibody-only control were included. After staining, samples were acquired using a BD LSRFortessa Cell Analyzer and the BD FACSDiva acquisition software (Becton, Dickinson and Company). Post-acquisition analysis was performed using the FlowJo Software (Becton, Dickinson and Company).

### Immunostaining

Cells were fixed with 4% paraformaldehyde in PBS for 20 minutes at room temperature and permeabilized in PBS with 0.5% Triton X-100 for 10 minutes. The staining was performed by incubating the primary antibody diluted in PBS + 10% heat-inactivated goat serum for 1 hour at 37°C. Slides were then washed in PBS three times and incubated with a secondary antibody conjugated to Cy-3 for 30 minutes at 37°C. Nuclear staining was performed by incubation with PBS + 40 ng/mL DAPI for 5 minutes. Slides were embedded with a DABCO/glycerol embedding solution and stored at 4°C until acquisition.

### Enzyme-linked immunosorbent assay

Human primary B cells were infected and cultured for the indicated amount of time. Cell culture supernatants were collected and analyzed for IL-6, TNFα, and IFN-α production using the Human IL-6 ELISABASIC kit (HRP) (Mabtech), the Human TNFα ELISABASIC kit (HRP) (Mabtech), and the Human IFN-α pan ELISABASIC kit (HRP) (Mabtech) following the manufacturer’s protocol.

### RNA isolation and cDNA synthesis

At the indicated time point, 2 × 10^6^ infected cells were pelleted and washed twice with ice-cold PBS 1×. Cell pellets were then lysed using the RLT buffer from the RNeasy Mini Kit (Qiagen). RNA isolation was performed on-column according to the manufacturer’s protocol. DNase treatment was performed on-column using the RNase-Free DNase Set (Qiagen) for 15 minutes at room temperature. RNA concentration and purity were assessed by Nanodrop quantification.

For cDNA synthesis, 250 ng of RNA was used to generate cDNA. The reaction was performed using random hexamers (for ZFP36L1, IL-6, TNFα, and TFRC) or specific primers (for EBNA2, Wp, EBNA1, EBNA3s, and GAPDH) and the AMV Reverse Transcriptase (New England Biolabs).

### Reverse transcription quantitative PCR

The reverse transcription quantitative PCR (RT-qPCR) and data analysis were carried out using the universal thermal cycling protocol on an ABI STEP ONE PLUS Sequence Detection System (Applied Biosystems). All RT-qPCRs included samples not treated with reverse transcriptase that served as negative controls. All samples were run in duplicate, together with primers specific for the human TFRC genes to normalize for variations in cDNA recovery.

The primers and probes used to detect ZFP36L1, IL-6, TNFα, EBNA2, Wp, EBNA1, EBNA3s, and TFRC are listed in Table S2. GAPDH quantification was performed using the Human GAPD (GAPDH) Endogenous Control primers and probe set (Applied Biosystems, 4310884E). For the reaction, the TaqMan Universal PCR Master Mix (Applied Biosystems) was used.

### Fluorescence *in situ* hybridization

For FISH, B cells were treated with an hypotonic solution containing 0.0075 m KCl, repeatedly fixed in ice-cold methanol:acetic acid (vol:vol 75:25) and applied onto glass slides. We generated an EBV-specific probe by nick translation of the complete B 95–8 BAC as described before (119). Denaturation of cells was performed in 2× saline-sodium citrate (SSC) 70% deionized formamide for 2 minutes at 75°C and followed by dehydration in an increasingly concentrated alcohol series. The probe was denatured at 75°C for 5 minutes, applied to dehydrated cells, and incubated overnight at 37°C. Slides were then washed in 4× SSC. The probe was detected by streptavidin-conjugated fluorescein Alexa 488 (Life Technologies). Slides were analyzed using a Leica epifluorescence microscope.

### Statistical analysis

All experiments were analyzed with either a paired *t*-test (one sample or two-tailed) or a one-way ANOVA with Tukey’s or Dunnett’s post hoc test. *P* values equal to 0.05 or less were considered significant unless otherwise indicated. The statistical analyses were performed with the GraphPad Prism 9 software (GraphPad Software). Mass spectrometry data were statistically analyzed using the software Perseus as described above.

## Data Availability

All relevant data supporting the findings of this study are available within the article and its Supplementary Information files, or from the corresponding author upon request.

## SUPPLEMENTAL MATERIAL

Supplemental material mbio.01784-23-s0001.docx Fig. S1-S7 and Tables S1-S3.

ASM does not own the copyrights to Supplemental Material that may be linked to, or accessed through, an article. The authors have granted ASM a non-exclusive, world-wide license to publish the Supplemental Material files. Please contact the corresponding author directly for reuse.
