# Supplementary material for: Epstein-Barr virus infectious particles initiate B cell transformation and modulate cytokine response
Source: mBio. 2023 Oct 13;14(5):e01784-23. doi: 10.1128/mbio.01784-23 (PMC10653912; doi:10.1128/mbio.01784-23)
Supplement: Supplemental material — Fig. S1-S7 and Tables S1-S3. [file mbio.01784-23-s0001.docx]

SUPPLEMENTARY MATERIAL FOR

**Epstein-Barr virus infectious particles initiate B cell transformation and modulate cytokine response**

Francesco Baccianti, Charlène Masson, Susanne Delecluse, Zhe Li, Remy Poirey & Henri-Jacques Delecluse

SUPPLEMENTARY FIGURES AND TABLES


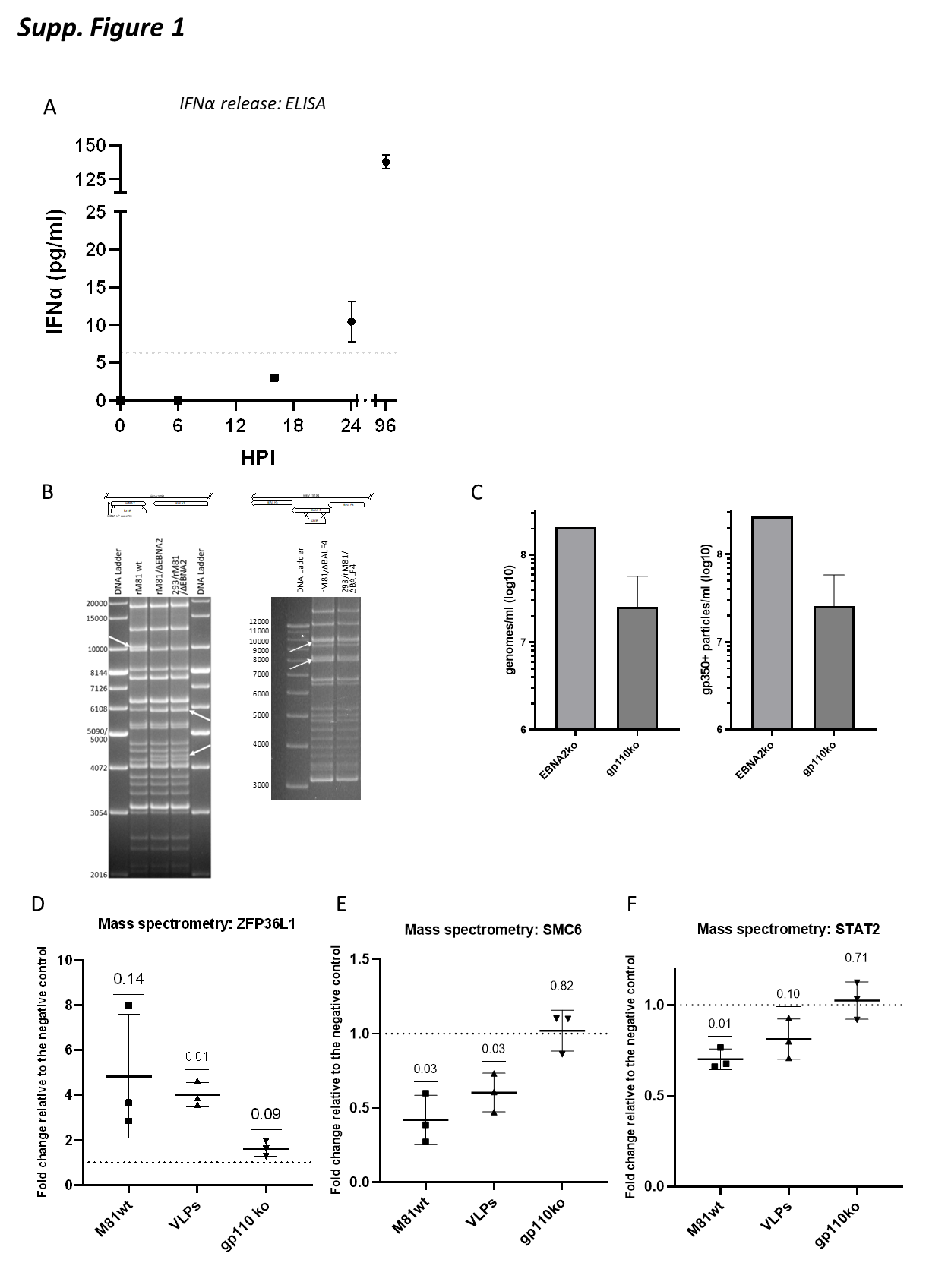


Suppl. Fig.1. Modulation of targets upon M81wt virus infection at the transcriptional and proteomic level. (A) Interferon α secretion was measured by ELISA at different time points after infection with M81wt virus of human primary B cells. The dotted line represents the lower detection limit of the kit used for the quantification as reported by the manufacturer. Two independent biological replicates were measured, and the data is shown as the mean ± SD. (B) Restriction analysis of the M81ΔEBNA2 and M81ΔBALF4 mutant virus DNA with BglII or BamHI, respectively, confirmed that BAC DNA from 293 producer cells generated the same restriction fragments as M81ΔEBNA2 and M81ΔBALF4 BAC DNA constructed in *E. coli*. White arrows emphasize DNA fragments that are different between wtEBV DNA and the mutants. (C) Quantification of viral genome containing particles (left) and gp350+ particles (right) produced by the EBNA2 and gp110 knockouts as quantified by qPCR or flow cytometry, respectively. The number of particles is reported per millilitre of supernatant. (D-F) Variation in the protein levels of ZFP36L1 (D), SMC6 (E), and STAT2 (F) compared to the negative control in M81wt virus, VLPs, and gp110 knockout virus-infected B cells at 6 hours post infection as identified by mass spectrometry. The fold change for each independent biological replicate is reported, and the mean ± SD is indicated. A one sample t test (μ=1) was performed. p values are reported above the comparison. p≤0.05 was considered statistically significant.

Suppl. Fig.2. Phosphoproteomic analysis of M81wt, VLPs and gp110 knockout infected B cells. (A-B) Volcano plot representation of significantly upregulated and downregulated phosphopeptides as identified by phosphoproteomic in 3 independent biological pooled replicates of primary human CD19^+^ B cells treated with M81wt VLPs (A) and gp110 knockout virus (B) at 6 hours post infection. A two-tailed paired t-test was performed in all cases. Targets were selected if their p value was ≤ 0.05 (horizontal dotted line) and the absolute fold change versus the negative control was > 2 (vertical dotted lines). Selected upregulated candidates for which the p value is above significance are shown in bold. (C) Euler diagram representing the events identified by phosphoproteomic as up- (left) or downregulated (right) in the three conditions as compared to the negative control. Only the events with a p value ≤0.05 and an absolute fold change >2 were considered. The name of the two upregulated phosphoevents that are identified in all three conditions is indicated. (D) Table listing the phosphorylated residues for the ZFP36 family members that were identified by mass spectrometry in the three conditions. Fold change and p values are reported in the indicated logarithmic scale. Values in red indicate a p value ≤0.05, while values in green indicate a fold change >2. (E) Top 10 enriched pathways as identified by pathway analysis performed on the phosphopetdides identified in the VLP-treated B cells. Predicted activation status is reported using different colours.


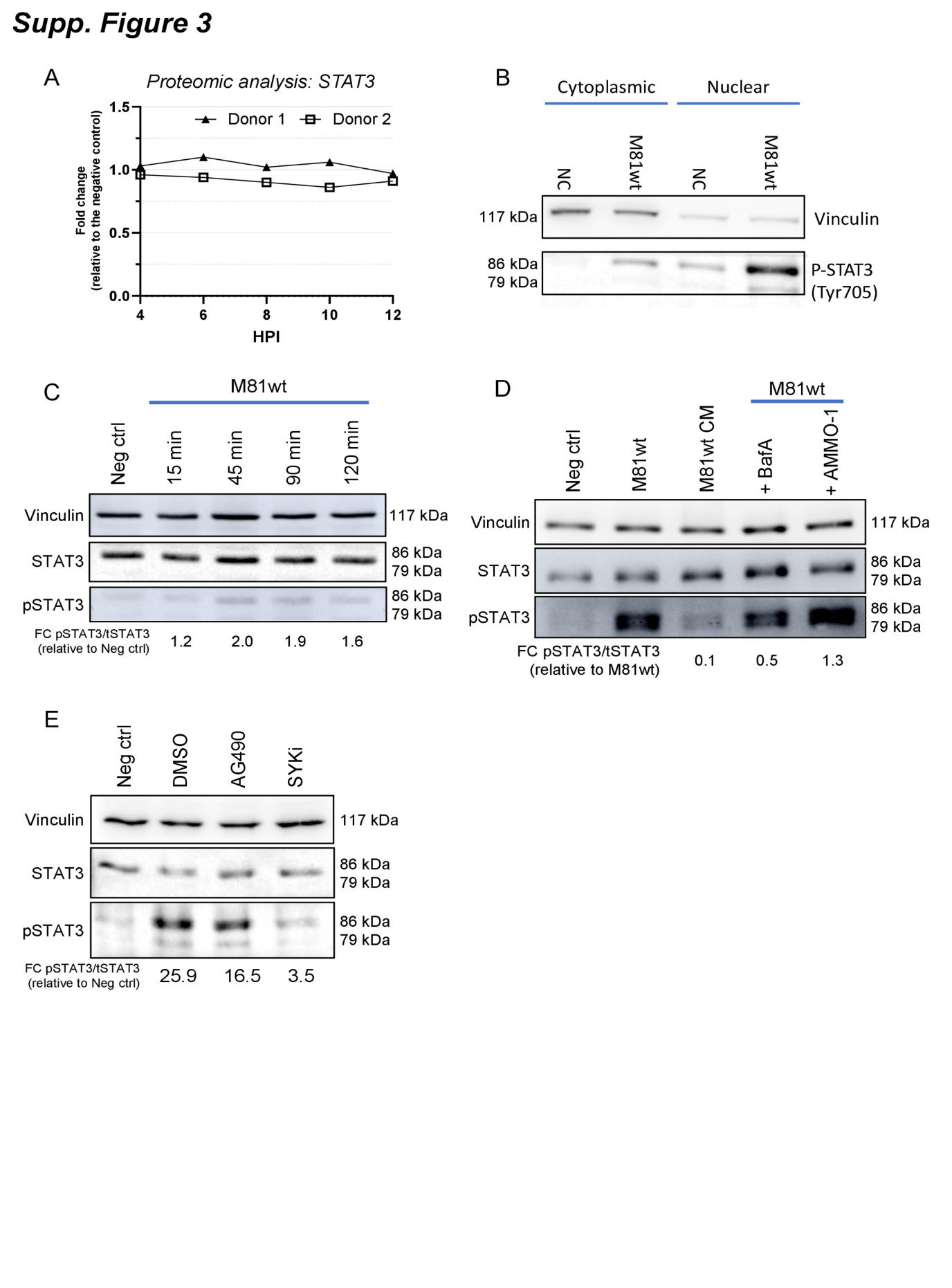


Suppl. Fig.3. Phospho-STAT3, but not total STAT3, is upregulated shortly upon EBV infection. (A) STAT3 protein levels as detected by mass spectrometry performed on two independent biological replicates of M81wt-infected B cells and expressed as fold change compared to the negative control at the different time points. The value of the fold change for each replicate is reported. (B) Cell fractionation performed on uninfected and M81wt-infected human primary B cells at 6 hours post infection. Nuclear and cytoplasmic fractions were isolated and activated STAT3 level was assessed. Vinculin was used as a marker of cytoplasmic contamination. (C-E) Western blot analysis for activated STAT3 (Tyr705) was performed on human primary CD19+ B cells (C) infected with M81wt at different time points shortly after infection; (D) infected for 6 hours with M81 virus alone, treated with conditioned medium obtained from M81wt supernatant, or infected with M81wt virus either in presence of bafilomycin A or after preincubation with the AMMO1 neutralizing antibody; (E) infected for 6 hours with M81wt virus in presence of the vehicle only (DMSO), the JAK2 inhibitor AG490, or the SYK inhibitor. (C-E) Total STAT3 and phospho-STAT3 (Tyr705) levels were assessed, and vinculin was used as loading control. Each blot is representative of at least 3 biological replicates and the reported values represent the fold change (FC) versus the indicated control for the normalized ratios of phospho-STAT3 over total STAT3.


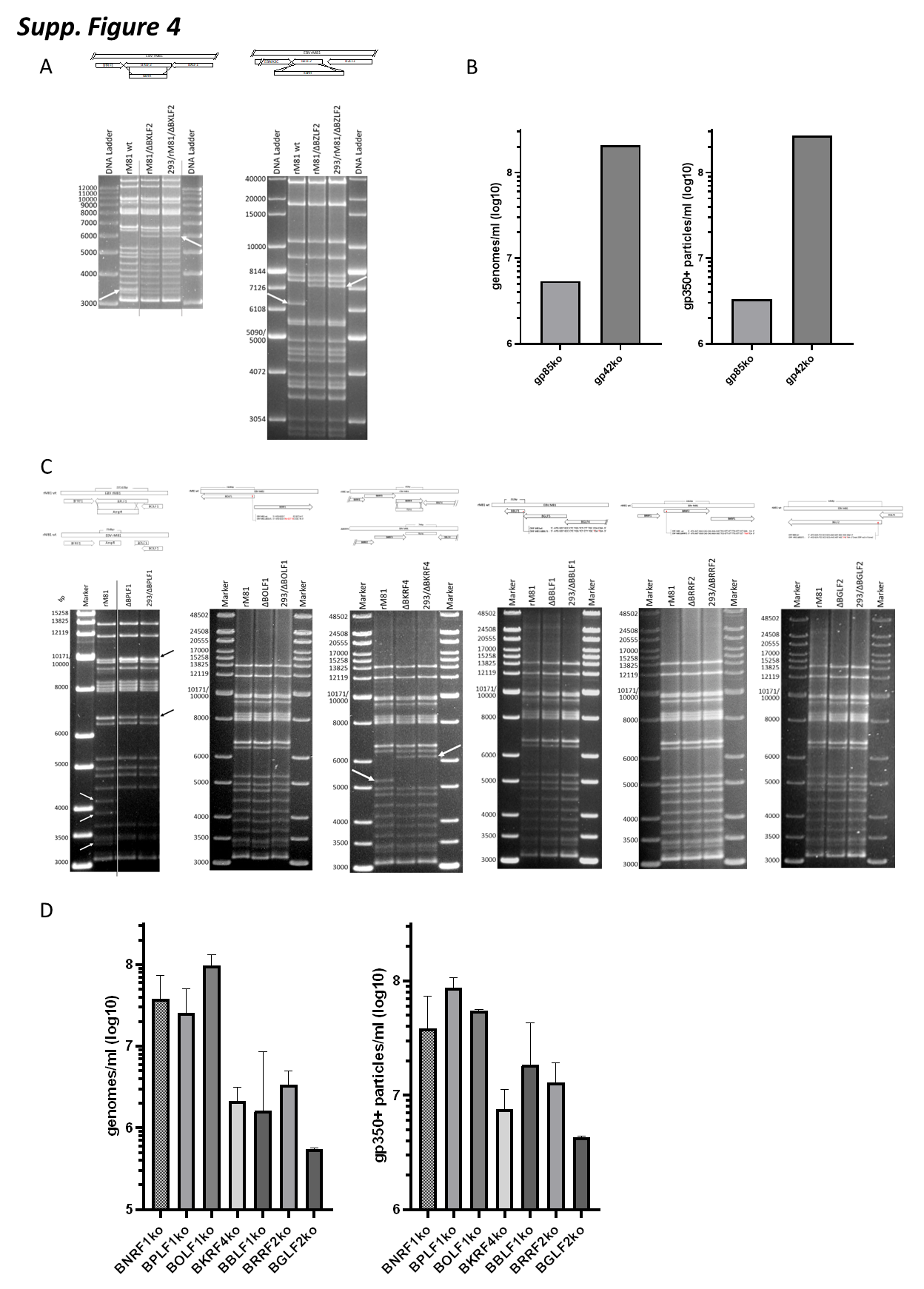


Suppl. Fig.4. Characterization of the EBV gp42, gp85, and tegument knockouts. (A) Restriction digestion of the M81Δgp85 and M81Δgp42 mutant virus DNA with BamHI or Eco91I, respectively, confirmed that the BAC DNA from 293 producer cells generated the same restriction fragments as M81Δgp85 and M81Δgp42 BAC DNA constructed in *E.coli*. White arrows emphasize DNA fragments that differ between wtEBV DNA and the mutants. (B) Quantification of viral genome containing particles (left) and gp350+ particles (right) produced by the gp85 and gp42 knockouts as quantified by qPCR or flow cytometry, respectively. The number of particles is reported per millilitre of supernatant. (C) Restriction analysis with BamHI confirmed that the BAC DNA from the different tegument knockout 293 producer cells generated the same restriction fragments as the respective BAC DNA constructed in *E.coli*. White and black arrows emphasize DNA fragments that are different in size between wtEBV DNA and the mutants. (D) Quantification of viral genome containing particles (left) and gp350+ particles (right) produced by the various tegument knockouts as quantified by qPCR or flow cytometry, respectively. The number of particles is reported per millilitre of supernatant.

**
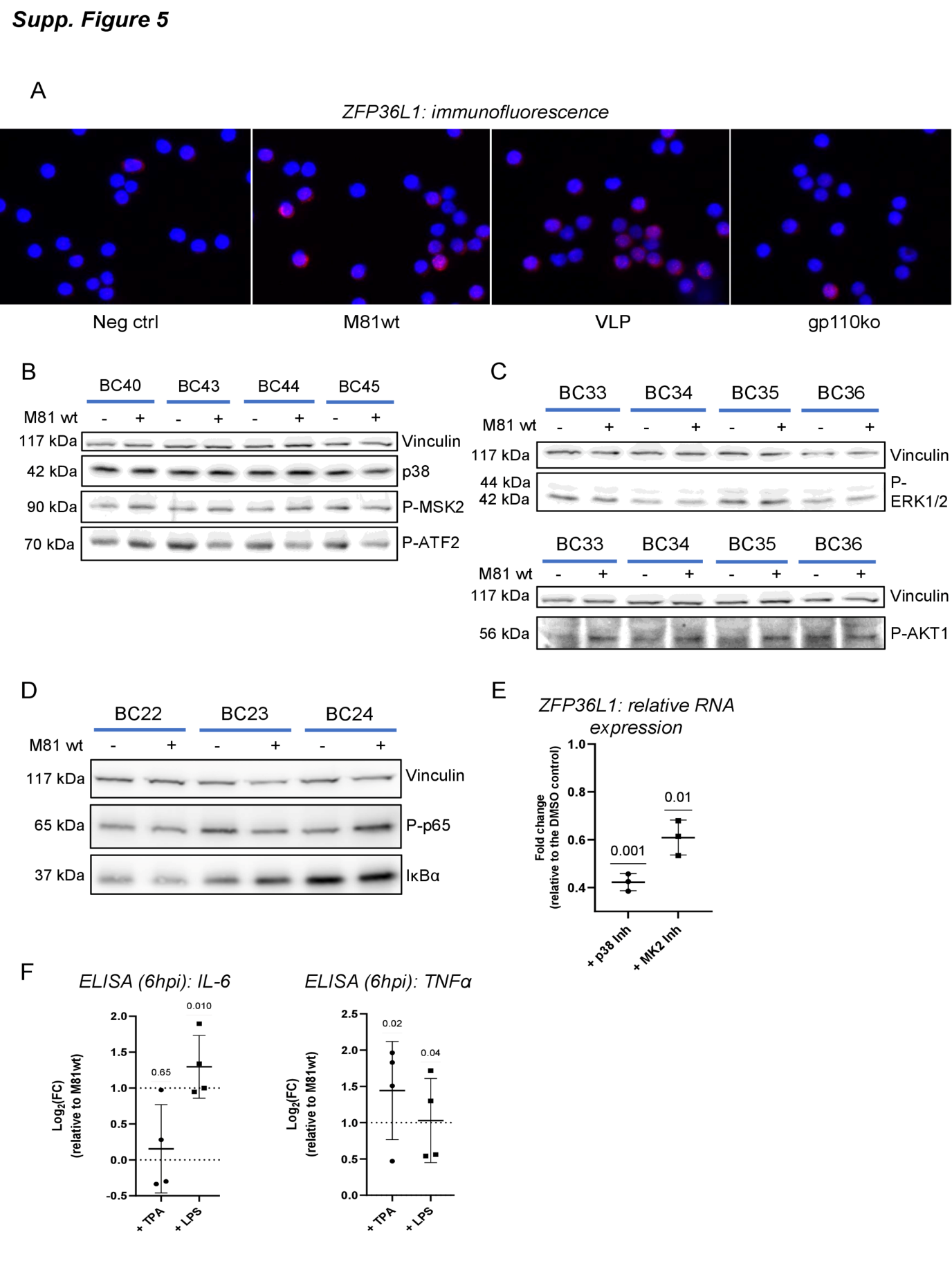
**

**Suppl. Fig.5.** **Activation status of several signaling pathways upon EBV infection.** (A) Immunofluorescence staining of primary human CD19^+^ B cells untreated or treated with M81wt, M81 VLPs or gp110 knockout virus at 6 hours post infection. Cells were stained for ZFP36L1 (red). Nuclei were counterstained with DAPI (blue). (B) Total p38 levels, as well as MSK2 and ATF2 activation status (Ser196 for MSK2 and Thr71 for ATF2) were assessed in B cells at 6 hours post infection with M81wt virus in 4 independent biological replicates. (C) ERK1/2 and AKT activation status was measured by western blot at 6 hours post infection with M81wt virus in 4 independent replicates by detection of phospho-ERK Thr202/Tyr204 and of phospho-AKT1 Ser473. (D) Phospho-p65 and total IκBα levels were investigated in 3 independent donors at 6 hours post infection with M81wt virus. Vinculin was used as loading control in all blots (B-D). (E) ZFP36L1 mRNA expression was evaluated by RT-qPCR on B cells infected for 6 hours with M81wt in presence or not of the p38 or MK2 inhibitors. Values are shown as the fold change between the treated and the untreated control sample for each independent replicate, and the mean ± SD is reported. (F) Quantification of IL-6 and TNFα secretion by ELISA in M81wt infected B cells at 6 hours post infection untreated or treated with TPA or LPS (n=4 independent biological replicates). The Log_2_(Fold change) is calculated relative to the untreated M81wt infected B cells. (E-F) A one sample t-test (μ=0) was performed. p values are reported above the comparison. p≤0.05 was considered statistically significant.

**Suppl. Fig.6.:** **Several pathways are involved in the regulation of EBV-induced IL-6 and TNFα secretion.** In order to investigate the role that the different pathways have on the secretion of IL-6 and TNFα upon EBV stimulation, IL-6 and TNFα levels were quantified in conditioned medium from primary B cells that were infected for 6 hours with M81wt virus in the presence of (A) BCR signaling inhibitors or (B) p38, MK2, PI3K, or NF-κB inhibitors. (C) The same experiment as in (A) was performed using the gp110 knockout virus to treat the cells. Values are expressed as Log_2_(Fold change) relative to (A-B) M81wt or (C) gp110 knockout infected cells. 3 (A, C) or 5 (B) independent biological replicates were analysed. A one sample t-test was performed (μ=0). p values are reported above the comparison. p≤0.05 was considered statistically significant. An asterisk is used to indicate when samples were below the detection limit and could not be quantified.

**Suppl. Fig.7: BGLF2 is required for nuclear transfer of the EBV episomes.** (A) Cell viability was measured for primary B cells infected with M81wt virus in the presence of various pathway inhibitors at 16 hpi with the alamarBlue viability reagent. The fluorescence emission measured at 590 nm is reported relative to the vehicle only control for each replicate and the mean ± SD is also shown (n=5 independent biological replicates). p values are indicated with a star notation (*, p≤0.05; **, p≤ 0.01; ***, p≤0.001). p≤0.05 was considered statistically significant. (B) Fluorescent *in situ* hybridization (FISH) was used to detect EBV episomal DNA in freshly M81wt or BGLF2 knockout infected CD19+ B cells (3 hours post infection). The percentage of cells showing nuclear episomes is reported, with each single replicate being shown, as well as the mean ± SD. 2 independent biological replicates were analysed. (C) EBNA2, EBNA1, EBNA3A, EBNA3B, and EBNA3C mRNA levels were quantified using RT-qPCR in two independent donors at 6 hours post infection with the M81wt virus and in uninfected primary B cells (negative control) or in an independently established LCL at 30 days post infection (positive control). The Ct value for each transcript in each sample is reported. The lack of amplification and detection in the sample for a transcript is reported as undetermined.

Supplementary Table 1. List of detected phospho-events which are upregulated (fold change>2), statistically significant (p value≤0.05) and shared by different conditions.

|  | Protein | Position |
| --- | --- | --- |
| Events in common to all three conditions | PPP1R18 | 368;368;43 |
|  | RBM7 | 205;84;204 |
| Events in common to M81wt and VLP | AIM1 | 892;484 |
|  | MON1B | 59 |
|  | DOCK2 | 1685;1177 |
|  | PRRC2C | 2107;2105;2058;1862 |
|  | JUND | 90 |
|  | SON | 94 |
|  | DYNC1I2 | 81 |
|  | FAM134C | 26 |
|  | DDX42 | 754;635 |
|  | CDC23 | 562;444 |
| Events in common to M81wt and gp110ko | CDS2 | 23 |
|  | STAT3 | 704,705 |
|  | MAP2K4 | 80;91 |
| Events in common to VLP and gp110ko | ZFP36 | 192;203;186 |
|  | ZFP36L2;ZFP36L1 | 490;334 |
|  | CXCR4 | 321;325 |
|  | TAF9 | 149 |
|  | PRKAG2 | 78;122 |
|  | ACIN1 | 400;342;360 |
|  | TRAPPC8 | 971 |

Supplementary Table 2. List of primers and probes.

| Internal # | Name | Sequence |
| --- | --- | --- |
| 1512 | EBNA2-pcp15 fwd | ACCTTCTAAGCACCCGCGCTTGTGTTTTGCTTTATCTGCCGCCATCAACAGCTATGACCATGATTACGCC |
| 1513 | EBNA2-pcp15 rev | GTAACATTTATTTGGGATACATTGGTTGCTGGAGAGGGCAACCAGTCACGACGtTGTAAAACGAC |
| 1647 | BALF4-kan fwd | CGCCTGGCACACCTCCGTCATCTTTTTTGTAAGGAAGATCAACAGCTATGACCATGATTACGCC |
| 1648 | BALF4-kan rev | ATGACTCGGCGTAGGGTGCTAAGCGTGGTCGTGCTGCTAGCAGTCACGACGTTGTAAAACGAC |
| 94 | Z2-Kan fwd | AAGTGAGCTAGATTAAGGGGATCCAAGGTGACCCCTGTTAACCAGTCACGACGTTGTAAAACGAC |
| 95 | Z2-Kan rev | AATAAATATCTTCCTGTCGGCTCTCTTTGAAGTGGTTGCCAACAGCTATGACCATGATTACGCC |
| 3431 | BXLF2 tet fwd | GTTTTTTGCCTGGTGTTGCTATGGGAGGTGGGGGCTGCCACTTCAAGATCCCCGATCTATGATTCCC |
| 2135 | BXLF2 tet rev | CATAACAATCTTGTGAACCAGAAAGATACCCAGAGCAAAAGCAATAAAGTCAGAGCGCTTTTGAAGCTCCAG |
| 2870 | BPLF1 amp fwd | TCCTAGGCACGGCCTCGTGCAACCAGGCCCACTGCAAGTTTGGCCGCTTTGCCGGCATCCTGTCAGAGGTTTTCACCGTCATC |
| 2871 | BPLF1 amp rev | GCACGTAAGAAACAGTAGAGGGCACGAAACATGGTGTATGCACTTTATTAATAAACAATTTCTACGGGGTCTGACGCTC |
| 2909 | BKRF4 kan fwd | TGAGAAGGGGCTCGGTGAGATAGATTGGAGGCTGTAGAGGGGTCATAACTACAGCTATGACCATGATTACGCC |
| 2910 | BKRF4 kan rev | ACTGAATAAAACAACAGACATGCAGACTCCAGGTTATGACATTTTATTTACCAGTCACGACGTTGTAAAACGAC |
| 3100 | BBLF1.insertion fwd | GCAGACAATACTTTTGACACCGCGCCATGGGTGCCCTCTGGTCTCTTTGCTGATGACGAGTCAACTCCTAGGGATAACAGGGTAATCGATTTATTCAACAAAG |
| 3101 | BBLF1.insertion rev | ATACAGGTTGATTATTCCCCCGTCCACGTCGCCTATGGAGTTGACTCGTCATCAGCAAAGAGACCAGAGGGTGCCAGTGTTACAACCAATTAACC |
| 3126 | BRRF2.insertion fwd | CATCGCTTAAGTATGAGTGGGCAGCAGAGAGGCTCGGTTATTTTGGTTCCTTGATCATCTGGCTGGGGCATTAGGGATAACAGGGTAATCGATTTATTCAAC |
| 3127 | BRRF2.insertion rev | TGTCCTGTGATAAAATCGCTCATAAGCTTAGTTAATGCCCCAGCCAGATGATCAAGGAACCAAAATAACTGCCAGTGTTACAACCAATTAACC |
| 3227 | BGLF2.insertion fwd | GGCCAATTGGCACTCACCCCCGAGGAGAGGGGATATATTCTGGCACGTCACGGCATCCGCCGCGAACAGTAGCTAGTAACAGCTAGGGATAACAGGGTAATCGATTTATTCAAC |
| 3228 | BGLF2.insertion rev | GCGTAGAGGCATCGCTCAGCACCCAGAGGCACTCCTTGTTGAGGAACTTGCGAAGCTGTTACTAGCTACTGTTCGCGGCGGATGCCGTGCCAGTGTTACAACCAATTAACC |
| 335 | hGAPDH cDNA | GATCTCGCTCCTGGAA |
| 333 | W2W1 cDNA | CCTAGGCCCTGAAGG |
| 334 | EBNA2 cDNA | GCAAGATAGAATGTAGGCAT |
| 348 | EBNA1 cDNA | GTACCTGGCCCCTCGTCA |
| 3278 | EBNA3A rev | ACAACCGAGGTAGATGGGACTTC |
| 3279 | EBNA3B  rev | GGCTTGCTGTGCTCTGCTG |
| 3272 | EBNA3C  rev | GTCCCCCTGTCCTTCAAATGATTCC |
| 363 | W0 fwd | CGCCAGGAGTCCACACAAAT |
| 364 | W0 | GAGGGGACCCTCTGGCC |
| 342 | W probe | FAM-ACCGAAGTGAAGGCCCTGGACCAAC-TAMRA |
| 349 | EBNA2 fwd type1 | GCTTAGCCAGTAACCCAGCACT |
| 344 | EBNA2 rev | TGCTTAGAAGGTTGTTGGCATG |
| 343 | EBNA2 probe | FAM-CCCAACCACAGGTTCAGGCAAAACTTT-TAMRA |
| 369 | Y3 fwd | TGCCTGAACCTGTGGTTGG |
| 368 | K rev | CATGATTCACACTTAAAGGAGACGG |
| 372 | U probe | FAM-TCCTCTGGAGCCTGACCTGTGATCG-TAMRA |
| 3270 | EBNA3 fwd | CCTCTGGAGCCTGACCTGTG |
| 3271 | EBNA3 probe | FAM-CGCATCATAGACCGCCAGTAGACCT-TAMRA |
| 3845 | hTFRC fwd | AATCCTGGGGGTTATGTGGC |
| 3846 | hTFRC rev | GGTGATTTTCCCTGCTCTGAC |
| 4358 | hTFRC probe | JOE-AGGCTGCAACAGTTACTGGT-BHQ |
| 3473 | hIL-6 fwd | CTTCGGTCCAGTTGCCTTCTC |
| 3474 | hIL-6 rev | TTACATGTCTCCTTTCTCAGGGC |
| 3587 | hIL-6 probe | FAM-AATTCGGTACATCCTCGACG-TAMRA |
| 3475 | hTNF-a fwd | GCTGCACTTTGGAGTGATCG |
| 3476 | hTNF-a rev | GCTTGAGGGTTTGCTACAACA |
| 3583 | hTNF-a probe | FAM-GAGTGACAAGCCTGTAGCCC-TAMRA |
| 3417 | hZFP36L1 fwd | AGTTTAAAGCTCCTCCTCCCCC |
| 3418 | hZFP36L1 rev | TTTCTGTCCAGCAGGCAACC |
| 3419 | hZFP36L1 probe | FAM-CGCCCCAGCCCGGAGTCAGAAA-TAMRA |

Supplementary Table 3. List of knockouts and the approaches used to generate them.

| Internal # | Name | Description |
| --- | --- | --- |
| B110 | M81wt | Recombinant M81 wild-type virus. |
| B279 | M81 BZLF2-kan-KO | Recombinant M81 in which the BZLF2 (=gp42) CDS has been replaced by the kanamycin resistance cassette from pCP15. |
| B975 | M81 EBNA2-kan-KO | Recombinant M81 in which the EBNA2 CDS has been replaced by the kanamycin resistance cassette from pCP15. The kanamycin cassette was excised by recombination using flp sites. |
| B1001 | M81 BALF4(∆nts42-1730)-kan-KO | Recombinant M81 in which the nts42-1730 of the BALF4 (=gp110) CDS have been replaced with the kanamycin resistance cassette from pCP15. |
| B1050 | M81 BFLF1/BFRF1A-KO BBRF1-KO BALF4-kan-KO (=VLPs) | Recombinant M81 B1001 containing the following deletions: BFLF1∆nts-43/+1394; BFRF1A ∆nts1-45; BBRF1 ∆nts299-1237. |
| B1099 | M81 BNRF1(aa1-428.expressed)-kan-KO | Recombinant M81 in which the BNRF1 CDS has been disrupted by inserting the kanamycin cassette from pCP15 (aa 1-428 are expressed). The kanamycin cassette was excised by recombination using flp sites. |
| B1580 | M81 BPLF1(aa60-3147end) | Recombinant M81 in which the BPLF1 CDS has been disrupted by inserting an ampicillin resistance cassette to replace aa 60-3147 (end). |
| B1636 | M81 BOLF1(stop.codon.after.aa2)-KO | Recombinant M81 in which the BOLF1 CDS has been disrupted by inserting a stop codon after aa2 and inserting a frameshift. The next ATG is not in phase. |
| B1641 | M81 BKRF4-kan-KO | Recombinant M81 in which the BKRF4 CDS has been replaced by the kanamycin resistance cassette from pCP15. |
| B1651 | M81 BBLF1(2stop.codons.after.aa8)-KO | Recombinant M81 in which the BBLF1 CDS has been disrupted by inserting two stop codons after aa8. The next ATG is not in phase. |
| B1669 | M81 BRRF2(1stop.codon+shift.after.aa13)-KO | Recombinant M81 in which the BRRF2 CDS has been disrupted by inserting a stop codon after aa13 together with a frameshift. |
| B1672 | M81 BGLF2(ATG-->ACG+2stop.codons.after.aa8-KO | Recombinant M81 in which the BGLF2 CDS has been disrupted by replacing the ATG with ACG, and inserting two stop codons after aa8. The next ATG is not in phase. |
| B1789 | M81 BXLF2(aa19-686)-tet-KO | Recombinant M81 in which the BXLF2 (=gH) CDS has been disrupted by inserting a tetracycline resistance cassette to replace aa 19-686. |
